# Housing Sulfur in Polymer Composite Frameworks for Li–S Batteries

**DOI:** 10.1007/s40820-019-0249-1

**Published:** 2019-02-27

**Authors:** Luke Hencz, Hao Chen, Han Yeu Ling, Yazhou Wang, Chao Lai, Huijun Zhao, Shanqing Zhang

**Affiliations:** Centre for Clean Environment and Energy, Environmental Futures Research Institute, School of Environment and Science, Griffith University, Gold Coast Campus, Gold Coast, QLD 4222 Australia

**Keywords:** Lithium–sulfur battery, Sulfur cathode, Binder, Binding mechanism, Polymer composite frameworks

## Abstract

Extensive efforts have been devoted to the design of micro-, nano-, and/or molecular structures of sulfur hosts to address the challenges of lithium–sulfur (Li–S) batteries, yet comparatively little research has been carried out on the binders in Li–S batteries. Herein, we systematically review the polymer composite frameworks that confine the sulfur within the sulfur electrode, taking the roles of sulfur hosts and functions of binders into consideration. In particular, we investigate the binding mechanism between the binder and sulfur host (such as mechanical interlocking and interfacial interactions), the chemical interactions between the polymer binder and sulfur (such as covalent bonding, electrostatic bonding, etc.), as well as the beneficial functions that polymer binders can impart on Li–S cathodes, such as conductive binders, electrolyte intake, adhesion strength etc. This work could provide a more comprehensive strategy in designing sulfur electrodes for long-life, large-capacity and high-rate Li–S battery.

## Highlights

- The roles of binders in both sulfur host-based and sulfur host-free systems are considered for polymer composite frameworks in lithium-sulfur batteries.
- The applications of the existing and potential multifunctional polymer composite frameworks are summarized for manufacturing lithium-sulfur batteries.

## Graphical abstract

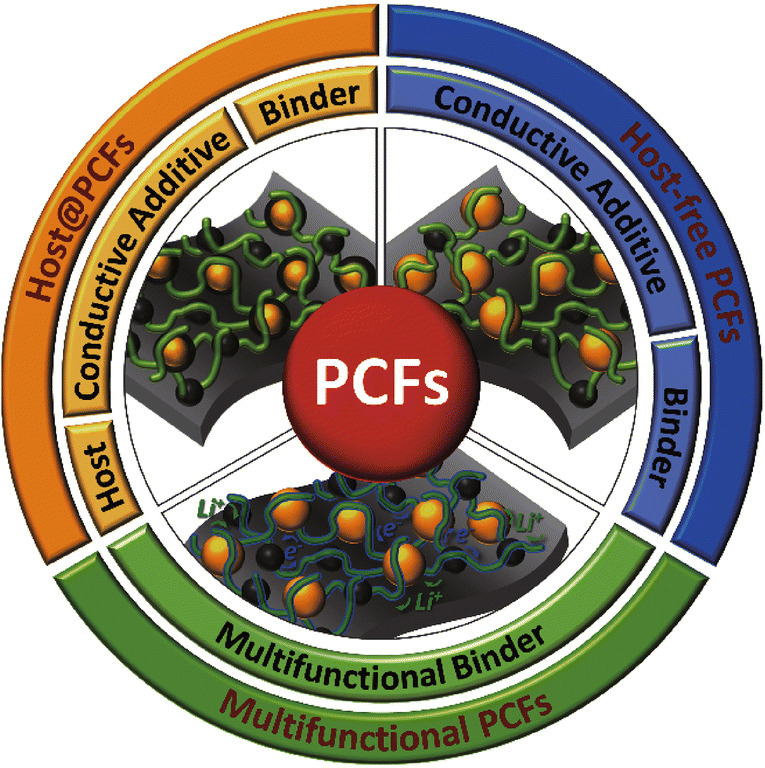

## Introduction

**Fig. 1:**
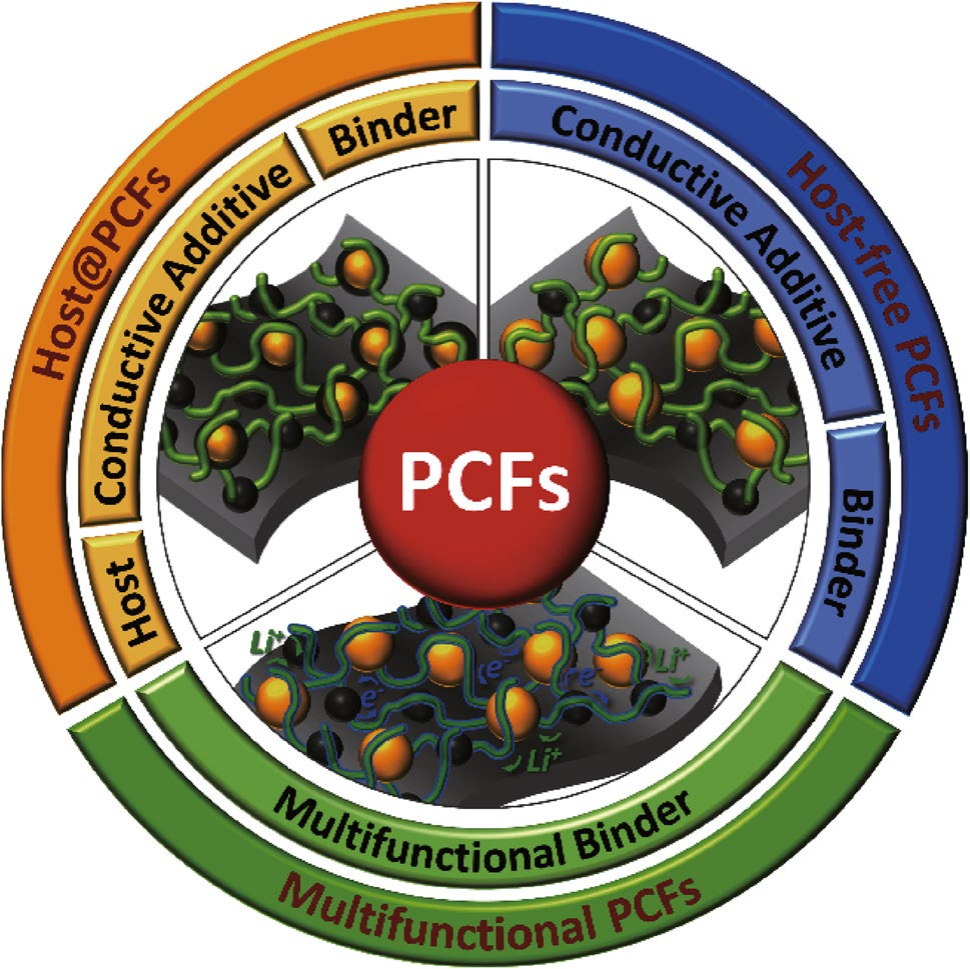
Three polymer composite frameworks (PCFs) in Li–S batteries; sulfur host@PCFs, sulfur host-free PCFs, and multifunctional PCFs for sulfur cathodes in Li–S Batteries

The recent increases in global population and worldwide development continue to put an upward pressure on the demand for energy [1]. As the majority of energy is still produced through the combustion of fossil fuels, this increased demand for energy continues to raise global greenhouse gas emissions, which is the driving force behind climate change [2]. To reduce the environmental impacts associated with society’s demands for energy, a transition away from fossil fuel-based energy to more renewable sources must be realized. With regard to grid scale energy generation, solar and wind power have made inroads into global energy infrastructure but are hindered by their intermittent energy supply [3]. A further uptake of electric vehicles (EVs) could also put downward pressure on the emissions arising from fossil fuel combustion for transportation, however for this to be realized on a larger scale, the travel range of EVs must be improved [4]. Current generation lithium ion batteries (LIBs) have been successfully applied in both grid scale energy storage as well as EVs, but the limitations of this mature technology are beginning to show. The high cost of LIBs is limiting their widespread application as grid scale storage devices, and their limited energy densities cap the travel range of EVs [5]. To counteract these shortfalls, researchers in the field are investigating more cost-effective and energy-dense rechargeable batteries. Lithium–sulfur (Li–S) batteries are a promising alternative to current generation LIBs, as their associated electrochemistry delivers an energy density up to 5 times higher than current cells [6]. Additionally, the active materials in Li–S cells are cheaper and more abundant than their traditional LIB counterparts. However, Li–S cells have hindrances in their commercial application due to their limited conductivity, volume expansion, and rapid capacity fading [7]. The most common method to address these concerns is through the rational design and implementation of nanostructured sulfur hosts, toward which a great deal of research has been focused [8]. In comparison, the design and implementation of novel polymeric binders has been largely overlooked in Li–S cells [9] and has only recently started to capture the attention of researchers, as shown in recent reviews [10–12], yet approaches investigating the entire cathode structure are lacking. To consolidate the current research in the field and provide future research directions, this review investigates the role of polymeric binders in the construction of polymer composite frameworks (PCFs). To begin with, we summarize the general binding mechanism in LIBs and then introduce the current challenges and solutions in Li–S batteries. Finally, we investigate the role of polymeric binders in host@PCFs, followed by a discussion on the role of binders in host-free PCFs, and finish with a review of multifunctional binders in PCFs, as shown in Fig. 1.

## Polymeric Binders in LIBs

### General Binding Mechanism

**Fig. 2:**
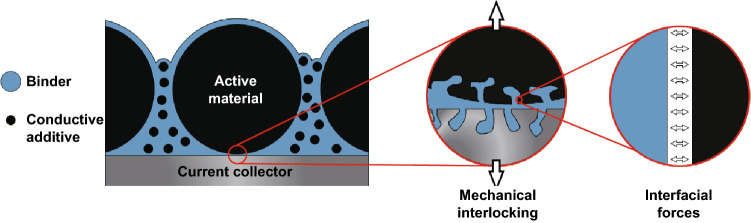
The roles of polymeric binders in a typical LIB

As the electrodes in LIBs are a composite electrode containing the active material and conductive additives, polymeric binders are employed to ensure intimate contact between the electrode components and the current collector is maintained over extended cycling. Before an in-depth review of binders in Li–S batteries is carried out, a brief summary of the adhesion mechanism is provided. Broadly speaking, an electrode slurry can be fabricated by combining a binder solution and the desired active materials. During this step, the solution can fully wet the surface pores of the particles. Once the slurry is coated and dried, adhesion throughout the polymer composite framework is achieved. This adhesion can be thought to arise via two mechanisms, mechanical interlocking and interfacial forces, as shown in Fig. 2 [13].

#### Mechanical Interlocking

D.E. Packham has provided an interesting history into the role of mechanical interlocking in adhesion theory [14]. Mechanical interlocking arises when a binder solution penetrates the pores of a particular surface (or surfaces) and is subsequently hardened. As the binder solution solidifies in situ a solid, embedded film remains in the pores of the material, leading to adhesion. The strength of this adhesion is influenced by the roughness of a particular surface, which allows for a higher area for bonding [15], as well as the nature of the adhesive itself [16].

#### Interfacial Interactions

Various adhesive mechanisms which occur at the interface between the adhesive and the active material surface have been proposed [17]. The mechanisms which are most commonly encountered in LIBs adhesion are those that include intermolecular forces, electrostatic forces, and covalent bonding which occur at the binder/surface interface. In the case of intermolecular forces, the adhesive strength between two materials can be improved if the ever present Van der Waals forces are supplemented by hydrogen bonding between the binder and substrate [18]. Similarly, further improvements in adhesive strength can be achieved if either electrostatic [19] or covalent bonds [20] occur at the interface. For a more comprehensive introduction to the forces that occur both at the interface and within a binder itself we refer readers to our recent review, which investigates the matter more thoroughly [13].

### Challenges of Traditional Binders

**Scheme 1:**
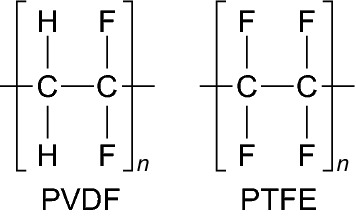
The molecular structures of the PVDF and PTFE

Fluorine containing polymers have experienced remarkable success when applied in energy storage devices such as batteries [13], supercapacitors [21], and fuel cells [22], and are the current status quo for binders in energy storage devices. Polyvinylidene fluoride or polyvinylidene difluoride (PVDF) (Scheme 1) is mainly produced by emulsion or suspension polymerization [11] and is the most widely used binder in battery electrodes due to its relative chemical inertness and stability over a wide voltage window [23]. Polytetrafluoroethylene (PTFE) (Scheme 1), another fluoro-polymer, has also found success in energy storage devices, particularly in supercapacitors due to its more superior tolerance to alkaline conditions compared with PVDF [24]. However, its inferior mechanical/adhesive properties have led to the dominance of the PVDF binder in battery systems. As PVDF is the most common binder in batteries, its limitations are the most relevant and are briefly discussed below.

#### Chemical Stability

Although wildly successful and chemically stable over a wide range of conditions, PVDF still causes operational concerns due to its chemistry. During high temperature operation, PVDF can react with lithium metal (or lithiated graphite) to form LiF [25]. Furthermore, under “abuse-conditions” such as over-charge/discharging, short circuits, etc., unwanted reactions with PVDF can cause thermal runaway which leads to safety concerns [26]. Finally, PVDF has been shown to cause accelerated degradation of active materials at contact points under elevated temperatures [27].

#### Adhesion Strength

The polymer backbone of PVDF consists of alternating CH_2_ and CF_2_ species which, according to the aforementioned binding theory, delivers adhesion through mechanical interlocking and Van der Waals forces. Although the C–F bond in PVDF is highly polar due to fluorine’s electronegativity, the polymer arranges itself so that the dipole moments cancel each other out [28]. Therefore, PVDF cannot produce strong interfacial interactions (i.e., hydrogen bonding, electrostatic interactions, or covalent bonds) toward the active materials or current collector, and, as a result, the stronger bonding mechanisms mentioned previously do not present themselves in PVDF-based electrodes. What is more, PVDF is prone to swelling in common LIB electrolytes, which can lead to the migration of the electrolyte between the binder/substrate interface [29], which reduces the intimate contact between electrode components required for strong bonding. Thus, it proves difficult for the PVDF binder to maintain a stable electrode structure over extended cycling.

#### Environmental, Health and Cost Concerns

PVDF is a rather costly synthetic polymer which is only soluble in volatile and toxic organic solvents, with the most commonly used solvent being *N*-methyl pyrrolidine (NMP) [30]. A shift toward aqueous-soluble binders could not only lower costs but could also reduce the associated health hazards and environmental impact associated with the manufacturing and recycling of secondary cells.

## Working Mechanisms and Challenges of Li–S Batteries

### Li–S Battery Working Mechanism

**Fig. 3:**
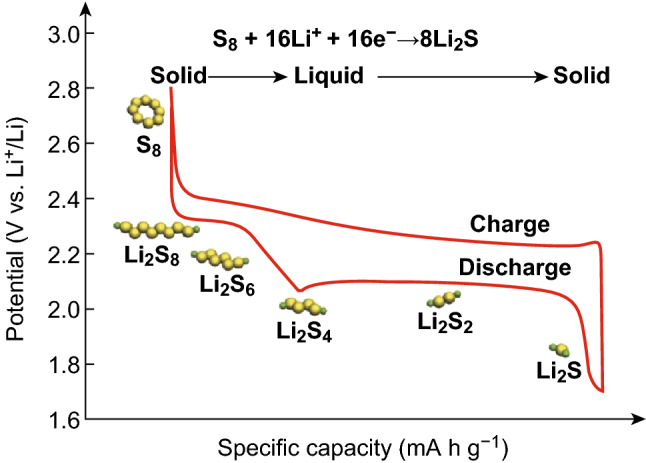
A typical charge/discharge profile for a Li–S battery. Reproduced with permission from Ref. [31]. Copyright 2017 John Wiley and Sons

A typical Li–S cell contains a composite sulfur cathode (containing sulfur, conductive additive, and binder), lithium metal anode, separator, and organic electrolyte. As discharge begins, Li^+^ ions migrate from the anode to the cathode so that the reduction of elemental sulfur can begin. A multi-step electrochemical reaction takes place with two associated voltage plateaus, as shown in Fig. 3 [31].

The voltage plateau at 2.4–2.15 V corresponds to the formation of long-chain polysulfides (Li_2_S_*x*_; *x* = 4–8). As the long-chain polysulfides are soluble in organic electrolyte, this portion of the electrochemical reaction involves a solid to liquid phase conversion of the active material and supplies ≈ 418 mAh g^−1^ toward the total discharge capacity [8]. Upon further lithiation, the long-chain polysulfides are converted to short-chain polysulfides (Li_2_S_*x*_; *x* = 1–2) which are insoluble in the electrolyte and precipitate at the cathode surface, providing the remaining ≈ 1254 mAh g^−1^ for a total of 1672 mAh g^−1^ specific capacity which roughly corresponds to an energy density of 2600 Wh kg^−1^ (based upon the complete formation of Li_2_S) [31].

### Challenges and Strategies of Li–S Batteries

The limitations of conventional LIBs have led researchers to investigate higher energy density storage options [6]. Li–S batteries, one of the most promising options, have received well deserved attention, with over 1000 research papers published on this topic since 2015 [9]. Such devoted attention to this system is aimed at solving the inherent problems with the Li–S cell, which are briefly introduced below.

#### Low Electronic and Ionic Conductivity of Sulfur

It is well established that sulfur cathodes suffer from low electron and ion transportation due to the insulating nature of both sulfur and its discharge product, Li_2_S, which results in poor rate kinetics and low sulfur utilization [32]. What is more, upon discharge a passivating layer of Li_2_S can form on the cathode surface, further reducing the cell’s capacity [33]. In Li–S cells, the low conductivities are typically addressed through the implementation of conductive sulfur hosts within the cathode [34, 35]. Additionally, to gain a better theoretical understanding of the ionic transport mechanisms within battery components, researchers have turned to DFT calculations [36].

#### Volume Expansion of Sulfur

Another challenge in Li–S cells pertains to the volume expansion experienced by the active materials during discharge [37]. Upon complete lithiation, the elemental sulfur undergoes a volume expansion of ≈ 70% [38], which can cause internal stresses within the electrode and results in electrode pulverization and capacity decay [39]. The volume fluctuations of the active materials are typically combated by the rational design and implementation of nanostructured sulfur hosts in the Li–S cathode [5].

#### The Shuttle Effect of Polysulfides

The most significant challenge relating to Li–S batteries is dubbed the “shuttle effect” (or shuttle phenomenon) [40]. This issue arises from the phase transformation of the active material that takes place during discharge, wherein the solid elemental sulfur is reduced to long-chain polysulfides (PSs) which are highly soluble in the common organic electrolytes found in Li–S cells. This formation of soluble long-chain polysulfides causes a concentration gradient to arise, which promotes the migration of these species toward the anode where they can undergo parasitic reactions, causing serious reduction in the discharge capacity and efficiency of the battery [41].

Initial attempts to curtail the PS shuttle involved the restriction of PSs through physical means, including surface coatings and the loading of sulfur into porous materials at the cathode, but more recent solutions include the chemical restriction of PSs [42]. Density functional theory (DFT) calculations have been utilized to investigate mechanisms by which PSs can be chemically anchored within the Li–S battery, including through the lithium bond [43], heteroatom-doping (particularly N and O doping) [44], and transition metal sulfide bonding [45].

A wide variety of materials have recently been investigated which aim to suppress the PS shuttle by various means, as summarized in the recent reviews on polar materials [46], metal oxide/sulfides [7, 47], organosulfur polymers [48], porous organic polymers [49], redox mediators [50], and flexible materials [51] for Li–S batteries. Most commonly, these chemical PS anchors are applied in the cathode of Li–S batteries which greatly improve Li–S performance [52–62]. Another successful approach to anchor the PSs and prevent their migration to the anode is through the use of functionalized interlayers and separators [34, 35, 49, 63–67]. Overall, anchoring the PS at either the cathode or the separator has greatly improved the capacity retention of Li–S cells over extended cycles.

#### Low Sulfur Loading and High Electrolyte/Sulfur (E/S) Ratio

There are two key considerations which must be addressed with regard to the sulfur loading in Li–S cells. The first relates to the sulfur weight fraction in the composite electrode and the second relates to the areal sulfur loading. Fang et al. [31] suggest a sulfur weight fraction of over 70% in the active materials and an areal loading of over 5 mg cm^−2^ for a reliable Li–S cell. Over the last few years, great improvements have been made with respect to both the sulfur loading in the composite and the areal sulfur loading [34].

The electrolyte is another crucial component of the Li–S cell. The go to solution for electrolytes in Li–S batteries has been liquid organic electrolytes [68], but recently researchers have turned their attention to solid electrolytes [69]. However, regardless of the electrolyte system chosen, another challenge with the Li–S cell is the excessive amount of electrolyte used in the cells reported in the literature, resulting in a high electrolyte to sulfur (E/S) ratio in reported test cells. Oftentimes, an E/S ratio that is greater than 7 µL of electrolyte to 1 mg of sulfur is used to obtain a high sulfur utilization; however, an E/S ratio of less than 4:1 is required so that the energy density of the Li–S cell can reach suitable levels [70]. Fang et al. [31] has shown that among literature which reports the E/S ratio of Li–S cells (which is already the minority of literature), over half use an E/S ratio of greater than 10:1, with only 3 achieving a ratio of 4:1 or lower. Liu et al. [34] has shown that little has changed with regard to the E/S ratio of Li–S cells in the literature over the past few years, more recently however, researchers are beginning to work on this problem [71].

#### Unstable Lithium Metal Anode

As mentioned earlier, the Li–S cell relies on a lithium metal anode, which is an attractive candidate for high-energy density batteries due to its superb theoretical capacity of ≈ 3860 mAh g^−1^ and low electrochemical potential of − 3.040 V versus the S.H.E. [72]. However, the Li metal anode suffers from a practically infinite volume expansion, parasitic reactions with the organic electrolyte and PSs, as well as dendrite formation during cycling, resulting in an unstable solid electrolyte interphase (SEI) layer, electrolyte depletion, and a decreased cycling efficiency [73, 74]. Attempts to rectify the problems caused by the lithium metal anode include the use of polymer protecting layers and artificial SEI layers, applied either ex situ, or formed in situ through the use of electrolyte additives [75]. Additional approaches include the fabrication of 3D host materials to house lithium metal [34, 35].

#### Safety of the Li–S Cell

In addition to the challenges regarding the performance of the Li–S cell, there are some significant safety concerns which must be overcome for successful Li–S commercialization. In addition to reducing cell efficiency, dendrite growth in Li metal anodes can pierce the separator and cause short circuits within the cell, resulting in thermal runaway and explosions [73]. Additionally, LiNO_3_ is commonly used as an electrolyte additive to passivate the Li anode and inhibit PS shuttling; however, it is prone to extreme gassing in larger pouch cells [70], causing an increase in internal pressure resulting in a risk of explosion [34, 69]. Recent approaches, which are aimed at increasing the safety of Li–S cells, include the application of specially tailored liquid and solid electrolytes [69] as well as the inclusion of flame-retardant materials within the cell [76, 77].

#### Polymer Composite Frameworks in Li–S Batteries

As mentioned earlier, a significant amount of research on the Li–S system has been focused toward the cathode host materials; however, the polymer binder, a crucial component of a high-performance cathode, is comparatively under researched [9]. In order to review the research progress in this area, we firstly classify the type of PCF based on the components present in the cathode. For the sake of this review, we define a host@PCF as a cathode constructed using sulfur, a sulfur host, conductive additives, and a polymeric binder. We discuss the role of the binders in host@PCFs in Sect. 4. Another PCF forgoes the traditional sulfur-host entirely and sulfur cathodes are fabricated simply through the combination of sulfur, conductive additives, and a binder. In this review, we dub these cathodes as host-free PCFs. The role of polymeric binders in host-free PCFs are reviewed in Sect. 5. Finally, researchers have turned to multifunctional binders to impart additional features into the Li–S cathode, which we review in Sect. 6.

## Sulfur Host@Polymeric Composite Frameworks

### Mechanical Interlocking Between Binders and Sulfur Host

A selection of hosts and binders in host-based Li–S batteries are listed in Table 1. It can be seen that the most commonly used binders are PVDF and PTFE. Due to the inertness of the polymers, the interaction between these polymers and sulfur is weak, however, because the polymer can penetrate and interlock the pores of the sulfur host, a relatively stable structure is obtained and the electrode can still deliver a good electrochemical performance for Li–S batteries [78, 79].

**Table 1:** PCFs via pure interlocking binding mechanism for Li–S batteries

Binder	Sulfur host	References
PVDF	Nitrogen-doped porous carbon	[78]
PVDF	Flower-shaped porous carbon	[80]
PVDF	Monolithic carbon	[81]
PVDF	Nitrogen-doped carbon nanofiber	[82]
PVDF	Hollow carbon nanofiber	[83]
PVDF	Carbon nanocube	[84]
PVDF	Nitrogen-doped porous carbon	[85]
PVDF	Porous carbon layer	[86]
PVDF	Mesoporous carbon	[87–91]
PVDF	Polypyrrole	[92]
PVDF	Carbon nanotube	[93, 94]
PVDF	Ti_4_O_7_	[95]
PVDF	MnO_2_	[96]
PVDF	Co_9_S_8_	[97]
PVDF	Porous carbon aerogel	[98]
PVDF	Li_2_S/TiO_2_-impregnated hollow carbon nanofiber	[99]
PVDF	Ti_2_C	[100]
PTFE	Porous carbon nanosheets	[79]
PTFE	Carbon sphere	[101]

**Fig. 4:**
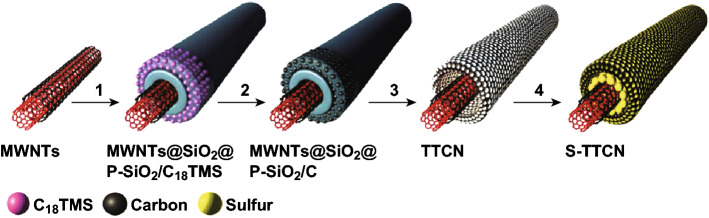
Schematic illustration for the formation of S‐TTCN composite: (1) Uniform coating a solid SiO_2_ layer and a porous SiO_2_ layer embedded with C_18_TMS molecules on MWNTs; (2) formation of porous carbon nanotube by carbonization of C_18_TMS; (3) etching SiO_2_ layers to obtain tube‐in‐tube carbon nanostructure (TTCN) with MWNTs encapsulated within hollow porous carbon nanotube; (4) sulfur infused into TTCN to fabricate S‐TTCN composite Reproduced with permission from Ref. [103]. Copyright 2014 John Wiley and Sons

As mentioned earlier, a vast array of sulfur hosts has been investigated for the use in sulfur cathodes. Of these, the carbonaceous host materials are normally porous so that the binder can mechanically interlock the sulfur host, while the host can provide an efficient confining structure for sulfur. Morphologies of the host can include hollow carbon spheres [102], carbon nanotubes [103], graphene [104], and hierarchical porous carbons [105]. For example, Zhao et al. synthesized a tube-in-tube carbon nanotube structure as a host for Li–S batteries while using. PVDF as a binder to fabricate a host@PCF structure, as shown in Fig. 4 [103]. Due to the good electrical conductivity and large pore volume of the porous carbon layers, the Li–S battery exhibited excellent electrochemical performance. The specific capacity still remained 918 mAh g^−1^ at 500 mA g^−1^ after 50 cycles and 647 mAh g^−1^ at 2 A g^−1^ after 200 cycles. It also delivered high capacity at high current density (550 mAh g^−1^ at 6 A g^−1^).

Similarly structured metal oxides [95], metal sulfides [97], and metal carbides [100] can provide sites for binder mechanical interlocking while simultaneously housing the sulfur-active materials. In-depth reviews on the design of sulfur hosts have already been provided by many researchers, which we direct readers to for further information [7, 34, 35, 46–51, 106–110].

### Combined Interfacial Forces in Polymer Composite Frameworks

In some cases, a host@PCF based on PVDF/PTFE can still deliver a good electrochemical performance due to the functionality provided by the sulfur host, while these binders simply maintain electrode integrity through weak adhesive interactions. However, to further improve the stability of the electrode, binders with functional groups have been explored for Li–S batteries, especially for high sulfur loading cathodes where PVDF/PTFE binders become insufficient. The binders poly(acrylic acid) (PAA), carboxymethyl cellulose (CMC)/styrene butadiene rubber (SBR), and sodium alginate (SA) contain abundant hydroxyl and/or carboxylate groups that are able to provide strong binding forces for the electrodes [13]. To obtain prolonged cycles for high-loading electrodes, the interaction between binders and hosts should be further enhanced. As shown in Table 2, many novel binders have been designed to synergistically work with the hosts.

**Table 2:** PCFs composed of binders and sulfur hosts with additional interfacial binding forces in Li–S batteries

Binder	Sulfur host	References
CMC/SBR	Meso@microporous carbon	[111]
CMC/SBR	Polypyrrole warped mesoporous carbon	[112]
CMC/SBR	Carbon nano fiber (CNF)	[113]
CMC/SBR	CNF	[114]
CMC/SBR	Co_9_S_8_	[97]
CMC	Polyacrylonitrile	[115]
CMC	Hollow porous carbon sphere	[116]
SA	Hollow carbon nanorod	[117]
SA	Microporous carbon	[118]
LA132	Nitrogen-doped carbon sphere	[119]
LA133	Core–shell carbon sphere	[120]
Poly(acrylic acid) (PAA)	S-CPAN	[132]
Poly(acrylonitrile-methyl methacrylate)	FeS_2_	[121]
Poly(vinylpyrrolidone) (PVP)/poly(ethylene oxide) (PEO)	CNF	[122]
Nafion/PVP	Porous carbon sphere	[123]
Nafion	Nickel sulfide/hollow carbon spheres	[124]
PVP	Porous carbon sheets	[125]
Poly(ethersulfone) (PES)	CNT	[126]
Poly[(*N*,*N*-diallyl-*N*,*N*-dimethylammonium) bis(trifluoromethanesulfonyl)imide] (PEB-1)	Nitrogen-doped mesoporous carbon	[127]
Poly[(2-ethyldimethylammonioethyl methacrylate ethyl sulfate)-*co*-(1-vinylpyrrolidone)] (D11)	Porous carbon sheets	[125]
Poly(diallyldimethylammonium triflate) (PDAT)	Porous carbon sheets	[125]
Polyaniline (PANi)	CNF/S	[133]
Guar gum (GG)	Poly(acrylonitrile) (PAN)	[128]
Carbonyl β-cyclodextrin (C-β-CD)	PAN	[129]
Polycation β-cyclodextrin (β-CDp-N^+^)	PANi	[130]
Double-chain polymer (DCP)	Carbon material	[131]

**Fig. 5:**
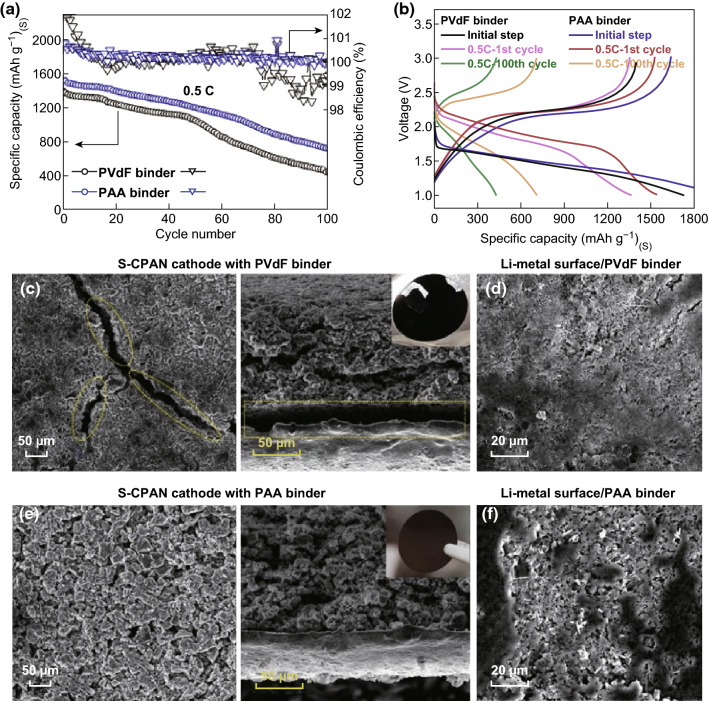
**a** Cycle retention graph and **b** charge–discharge curve graph of S-CPAN cells with PVdF and PAA binders. SEM images of **c** surface and cross section of S-CPAN with PVdF binder electrode, **d** lithium metal surface of S-CPAN cell with PVdF binder, **e** surface and cross section of S-CPAN with PAA binder electrode, and **f** lithium metal surface of S-CPAN with PAA binder after 100 cycles. Reproduced with permissions from Ref. [132]. Copyright 2017 American Chemical Society

For example, a PVDF binder was sufficient to maintain electrode integrity and obtain stable cycle performance when using a Co_9_S_8_ host with a sulfur loading below 2.5 mg cm^−2^. However, when the electrodes were fabricated with higher sulfur loadings (2.5–4.5 mg cm^−2^), the use of a CMC/SBR binder was required to maintain the high capacity and stable cycles [97]. Another example is that Kim et al. [132] investigated PAA as a binder in host@PCF Li–S cathodes. The group combined sulfurized carbonized poly(acrylonitrile) (S-CPAN) as a sulfur host and PAA as a binder to form the framework. The PAA-based electrode delivered a higher specific capacity upon cycling compared with the PVDF-based electrode, while also delivering a higher Coulombic efficiency (Fig. 5a, b). After 100 cycles, post-mortem analysis of the electrode cross section under SEM revealed severe delamination in the PVDF-based framework (Fig. 5c); however, there was still intimate contact between the electrode film and current collector when PAA was used as the binder (Fig. 5e). The surface of the PVDF-based electrode displayed large cracks, whereas the PAA-based electrode maintained its integrity. The group suggested that the structural integrity was maintained in the cathode due to hydrogen bonding occurring between the carboxylate groups of the PAA and the OH groups found on the carbonized PAN and current collector. This hydrogen bonding displays high elasticity and was able to maintain intimate contact between electrode components during the volume expansion/contraction of the S-CPAN upon cycling. Following this, an FEC additive was used to stabilize the lithium metal anode in the alkyl carbonate electrolyte, which enabled a capacity retention of 98.5% (≈ 1500 mAh g^−1^) after 100 cycles at 0.5C.

**Fig. 6:**
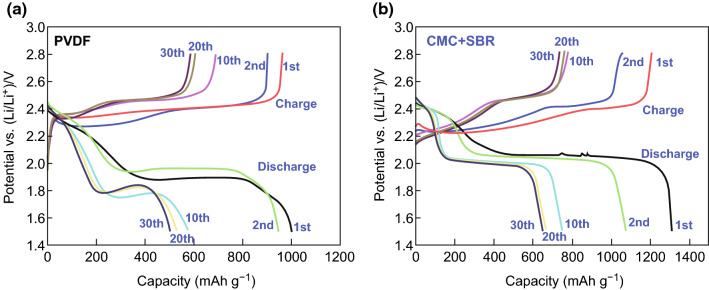
Charge and discharge curves of a lithium/sulfur cell with **a** PVDF binder and **b** CMC + SBR binder at 0.05C. Reproduced with permission from Ref. [114]. Copyright 2012 Elsevier

Rao et al. [114] used a chemical deposition method to prepare a CNF-S composite. From there, they fabricated host@PCF cathodes using PVDF in NMP, poly(ethylene oxide) (PEO) in acetonitrile, and CMC/SBR (2:3) in water as binders, respectively. By observing the discharge profiles (Fig. 6), it was seen that the CMC/SBR and PEO-based frameworks displayed a lower voltage plateau of around 2.0 V, compared with the PVDF-based framework’s lower voltage plateau of 1.95 V, which suggests a greater degree of polarization in the PVDF-based cell. Upon extended cycling, the discharge capacities were 586, 420, and 350 mAh g^−1^ for the CMC/SBR, PEO, and PVDF-based batteries, respectively, which highlighted the superior capacity retention when CMC/SBR is used as a binder.

**Fig. 7:**
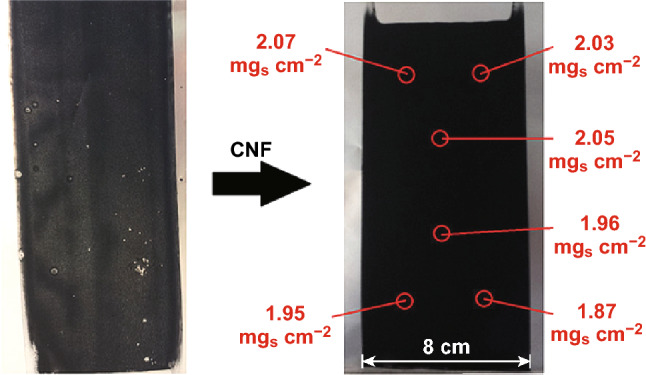
Photographs comparing the effect of a 3.5% w/w addition of carbon nanofibers to water-based slurries employing a PVP:PEO binder. Uniformity of sulfur loading is indicated for the coating with CNF. Reproduced with permission from Ref. [122]. Copyright 2017 Wiley‐VCH Verlag GmbH & Co. KGaA, Weinheim

Lacey et al. investigated the effects of binders and hosts in Li–S batteries. They fabricated an acceptably high sulfur loading cathode using their optimized polyvinylpyrrolidone (PVP):PEO binder [122]. They found that with a small addition of CNFs into the cathode, the homogeneity of the electrode film was vastly improved (Fig. 7), which allowed for an increased sulfur loading up to 5 mg cm^−2^ without delamination of the electrode film.

**Fig. 8:**
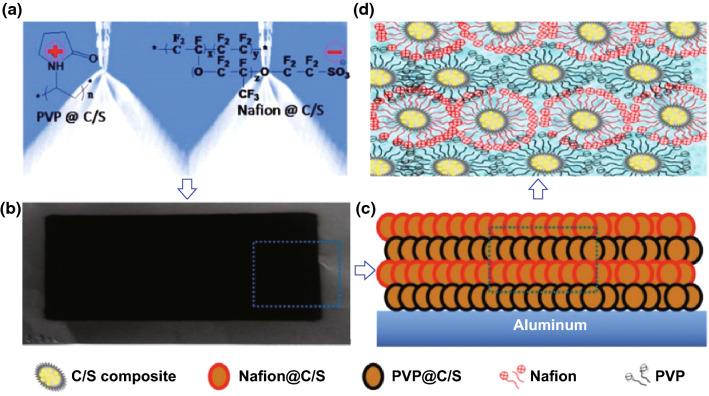
Illustration of **a** the air spray process of cathode, **b** the sprayed cathode, **c** the layer-by-layer C/S composite, **d** the cross-link between Nafion and PVP. Reproduced with permission from Ref. [123]. Copyright 2015 American Chemical Society

The mechanical properties of the electrodes can also be enhanced by oppositely charged binders. Soft-pack Li–S batteries with an ultra-low binder content of 0.5 wt% were fabricated by Wang et al. [123]. The group used an innovative layer-by-layer air spray method to synthesize a Nafion/PVP (N/P)-based Li–S electrode (Fig. 8). The adhesion of the electrode film was examined via a peel test, which revealed the N/P binder delivered a stronger adhesion with 0.5 wt% than the PVDF film with 10 wt% loading owing to the electrostatic interaction between the positively charged PVP and negatively charged Nafion. The as-fabricated pouch cells delivered a higher initial capacity and a slower capacity decay compared with the PVDF batteries, even with the ultra-low binder loading.

Besides the strong binding forces, the binders are also expected to be multifunctional. Considering that, various functional binders have been explored for host-based sulfur electrodes, such as electronically and ionically conductive binders [115, 131]. For example, a polypyrrole (PPy)-based double-chain polymer binder was developed by Liu et al. [131]. 4,4ʹ- Biphenyl disulfonic acid (BSA) was capped with pyrrole before being polymerized on a CMC matrix. The incorporation of 6.4 wt% of the BSA/PPy into the CMC matrix reduced the resistance of the composite, in turn greatly increasing the conductivity of the cathodes fabricated using this binder, while simultaneously providing anchoring sites for PS retention. A thick electrode with a sulfur loading of 9.8 mg cm^−2^ was fabricated and delivered a high areal capacity of 9.2 mAh cm^−2^ even with a low electrolyte to sulfur ratio of 5:1 (µL:mg).

**Fig. 9:**
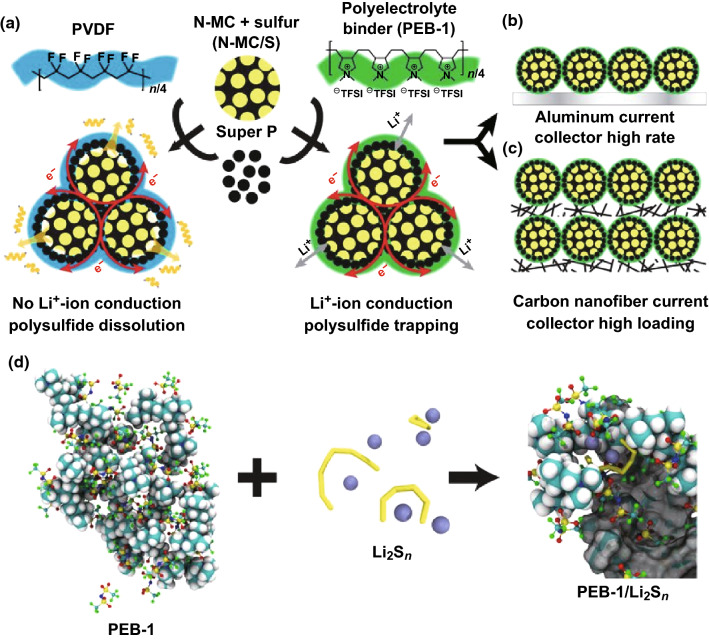
Illustration of the fabrication of sulfur electrodes with PVDF or PEB-1 binder. **a** The cathode is comprised of sulfur-active materials loaded into *N*-doped mesoporous carbon (N-MC) hosts, “Super-P” as the conductive additive, and a polymer binder (PEB-1 or PVDF). **b** A conventional sulfur cathode cast onto an aluminum current collector. **c** A highly loaded sulfur cathode cast onto a carbon nanofiber current collector. **d** Schematic illustrating the formation of complex ion clusters via anion metathesis, when PEB-1 encounters soluble polysulfides during Li–S cell cycling. Reproduced with permissions from Ref. [127]

Binders can also facilitate the ion transport across the electrolyte-host interface. Li et al. [127] introduced a polyelectrolyte binder—poly[(*N*,*N*-diallyl-*N*,*N*-dimethylammonium) bis(trifluoromethanesulfonyl)imide] (PEB-1) to sulfur cathode with nitrogen-doped mesoporous carbon (N-MC) hosts, as shown in Fig. 9. Due to the high ionic conductivity of PEB-1, the utilization of sulfur could be enhanced even in the depths of the mesoporous carbon. The Li–S batteries with high sulfur loading could deliver high capacities at a fast rate (1004 mAh g^−1^ at 0.2C with a high mass loading of 8.1 mg cm^−2^) and exhibit long cycle life, which is attributed to the large N-doped surface area of the N-MC and facile Li^+^-ion transport in the electrode as aided by PEB-1.

To further improve the design of hosts for sulfur cathodes, free-standing structures can be realized. A free-standing CNF/S/polyaniline (PANi) cathode was introduced by Zhu et al. [133]. A S/CS_2_ solution was first used to impregnate a carbon nanofiber mat with sulfur before a coating of PANi was applied through an in situ polymerization process. The resultant electrode delivered a reversible discharge capacity of 953 mAh g^−1^ after 300 cycles at 0.2C owing to the ability of the 3D architecture to accommodate the sulfur volume expansion/contraction during cycling. The energy density of the entire electrode was improved through the reduction in unnecessary electrode components.

Overall, the implementation of rationally designed sulfur hosts has made great strides in overcoming the technical challenges associated with Li–S cells. However, there has been comparatively little research into the cooperative effects realized by sulfur hosts and novel binders. Future progress could be made by further investigation into the combination of sulfur hosts and novel multifunctional binders.

## Sulfur Host-Free Polymeric Composite Frameworks

Sulfur-host-free PCFs are fabricated without the use of a traditional sulfur hosts. As there is no sulfur host present, it is the responsibility of the polymeric binders in host-free PCFs to provide a stable electrode structure. This section reviews the research progress on host-free PCFs in Li–S batteries. A table overviewing the performances of host-free PCFs and their respective binders is provided in Table 3.

**Table 3:** Binders and their electrochemical performance in host-free PCFs

Binder	Discharge capacity @ *n*th cycle	C-rate	References
*Natural polymers*			
Gelatin	544 mAh g^−1^ @ 50 cycles	≈ 0.1C	[134]
Sodium alginate	508 mAh g^−1^ @ 50 cycles	≈ 0.2C	[135]
CMC/SBR (1:1)	580 mAh g^−1^ @ 60 cycles	≈ 0.05C	[136]
Chitosan	≈ 950 mAh g^−1^ @ 20 cycles	0.1C (1st 3 cycles), 0.5C remaining cycles	[137]
Gum Arabic	841 mAh g^−1^ @ 500 cycles	0.5C	[138]
Guar Gum	≈ 600 mAh g^−1^ @ 400 cycles	1C	[139]
Carrageenan	≈ 700 mAh g^−1^ @ 100 cycles	0.05C	[140]
Starch	≈ 500 mAh g^−1^ @ 200 cycles	0.2C	[141]
*Synthetic polymers*			
Poly(vinylpyrrolidone)	≈ 1000 mAh g^−1^ @ 100 cycles	0.2C	[142]
Poly(ethylene oxide)	≈ 650 mAh g^−1^ @ 50 cycles	0.2C	[143]
poly(acrylamide-*co*-diallyldimethylammonium chloride)	652 mAh g^−1^ @ 100 cycles	≈ 0.05C (1st 6 cycles), ≈ 1C remaining cycles	[144]
Poly(acrylic acid)	325 mAh g^−1^ @ 50 cycles	0.2C	[145]
LA132	470 mAh g^−1^ @ 100 cycles	0.5C	[146]
Poly(amidoamine)	≈ 640 mAh g^−1^ @ 100 cycles	0.05C (1st 2 cycles), 0.2C remaining cycles	[147]
Poly(ethylenimine)	744.2 mAh g^−1^ @ 50 cycles	0.05C	[148]
Poly[bis(2-chloroethyl) ether-alt-1,3-bis[3(dimethylamino) propyl]urea] quaternized	885.1 mAh g^−1^ @ 50 cycles	0.05C	[149]
Poly(diallyldimethylammonium triflate)	≈ 700 mAh g^−1^ @ 50 cycles	0.1C	[125]
Polymeric ionic liquid 5	446 mAh g^−1^ @ 500 cycles	0.2C	[150]
Thiokol	501 mAh g^−1^ @ 200 cycles	0.1C	[151]
Ammonium polyphosphate	530 mAh g^−1^ @ 200 cycles	0.5C	[77]
*Composite binders*			
PVP:PEO (1:4)	≈ 1000 mAh g^−1^ @ 50 cycles	0.2C	[152]
PEI:PVP	≈ 580 mAh g^−1^ @ 50 cycles	0.1C charge, 0.25C	[153]
PEI:Gelatin	871.3 mAh g^−1^ @ 100 cycles	0.5C	[154]
*Cross-linked binders*			
SA/Cu^2+^	758 mAh g^−1^ @ 250 cycles	0.2C (1st cycle), 1C remaining	[155]
Xanthan gum/Guar gum	724 mAh g^−1^ @ 150 cycles	0.5C	[156]
Amino functional group binder	≈ 400 mAh g^−1^ @ 600 cycles	2C	[157]
PEI/poly(ethylene glycol) diglycidyl ether	430 mAh g^−1^ @ 400 cycles	1.5C	[158]
PEI/epoxy resin	829 mAh g^−1^ @ 1000 cycles	0.5C	[159]

### Natural Polymers

Natural polymers have been a staple in binder research since Kovalenko et al. [160] used alginate to fabricate high-performance silicon anodes in LIBs. Natural polymers are abundant, environmentally friendly, aqueous-soluble, and are endowed with a high degree of functionality via their inherent functional groups. As such, natural polymers are an attractive option when fabricating host-free sulfur cathodes.

#### Gelatin

Gelatin is a water-soluble biological macromolecule and in an aqueous solution, and it delivers a sufficient viscosity to function as a binder in rechargeable battery electrodes [161]. Huang et al. [162] applied gelatin derived from bovine bones to form a bio-derived host-free cathode in Li–S batteries. When compared with an electrode fabricated with PEO, it was observed that the gelatin-based cathode displayed superior homogeneity of the sulfur and acetylene black conductive additive. The –COOH and –NH_2_ functional groups contained in gelatin allowed for a high adhesion among the electrode components and current collector. Furthermore, as these functional groups are highly hydrophilic, the resultant polymeric framework was substantially insoluble in the organic electrolyte, which resulted in a superior performance of the gelatin-based cathode [161].

**Fig. 10:**
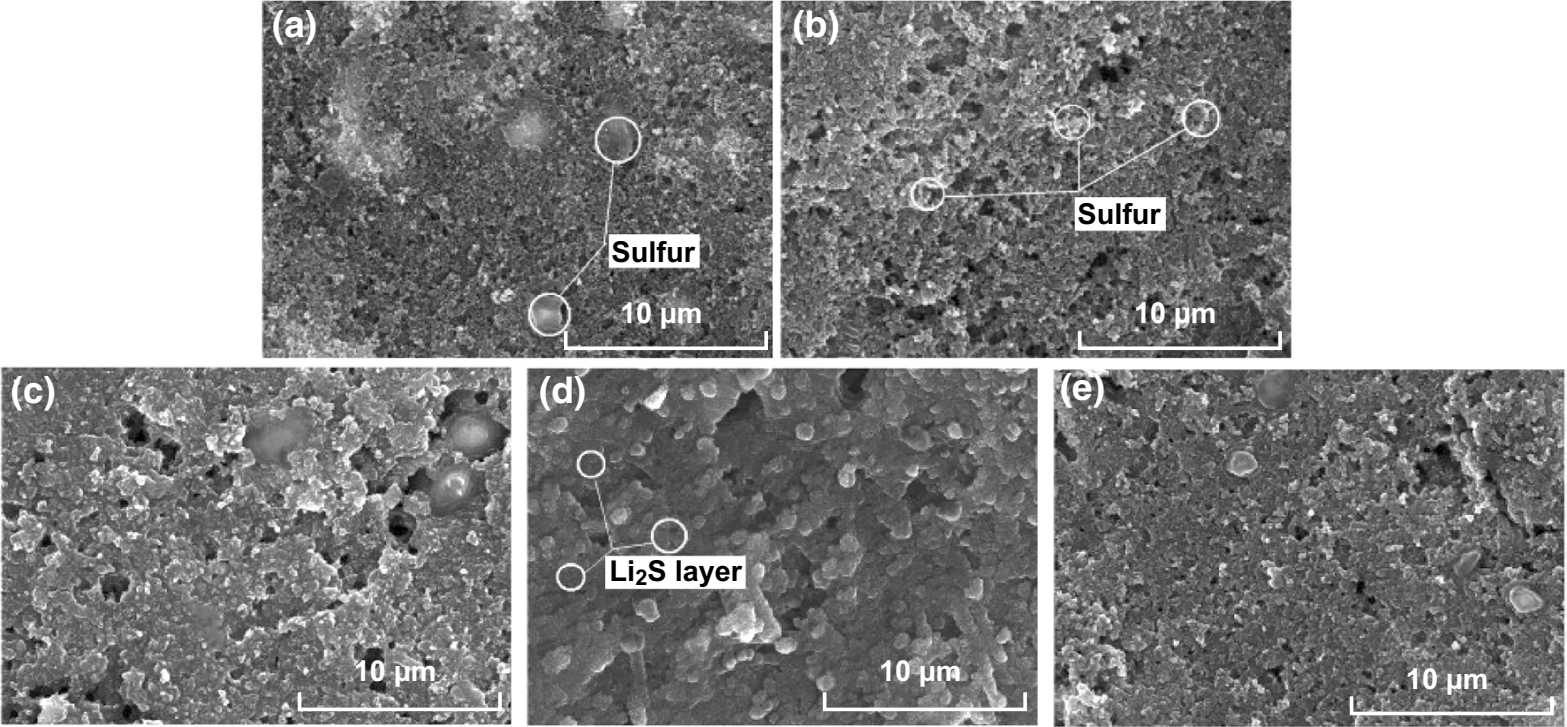
SEM images of the porous sulfur cathodes during the discharge–charge process at the **a** original, **b** 6% discharge, **c** 36% discharge, **d** full discharge and **e** first charge. Reproduced with permission from Ref. [163]. Copyright 2009 Elsevier

Wang et al. [163] characterized a gelatin-based sulfur cathode at different stages of discharge via SEM and XRD analysis. SEM images take prior to first discharge (Fig. 10a) reveals a homogeneous distribution of sulfur, carbon, and pores throughout the polymeric framework. Figure 10b reveals the reduction in pore volume as elemental sulfur is reduced to long-chain polysulfides, with Fig. 10c revealing a further reduction in pore volume as the long-chain polysulfides are further reduced to the insoluble short-chain polysulfides. Upon full discharge (Fig. 10d), the Li_2_S layer becomes denser with a further reduction in porosity across the electrode. After a full charge, the Li_2_S layer is fully oxidized and the porous structure of the framework returns (Fig. 10e). The gelatin-based cathode obtained a capacity of 1235 mAh g^−1^ at the first discharge and retained a capacity of 626 mAh g^−1^ after 50 cycles at a discharge current density of 0.4 mA cm^−2^, which the group attributed to the framework’s ability to retain a stable void structure after PS dissolution.

**Fig. 11:**
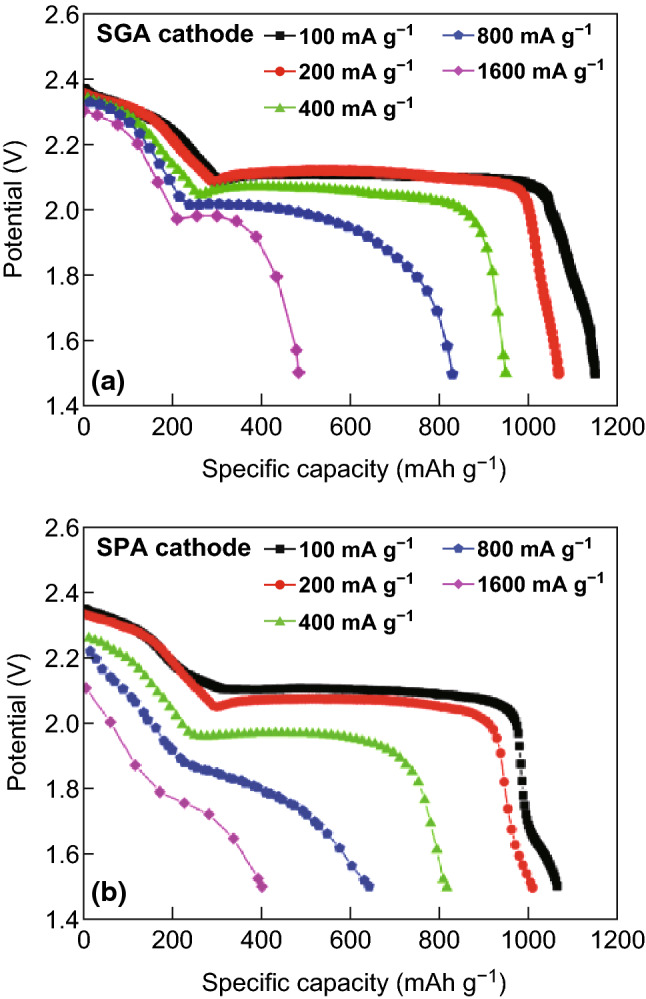
Initial discharge curves of Li/S batteries using SGA (gelatin-based) cathode (**a**) and SPA (PEO-based) cathode (**b**) at different current densities. Reproduced with permission from Ref. [134]. Copyright 2011 The Electrochemical Society

Following this, the group observed the electrochemical behavior of both the gelatin-based (SGA) and PEO-based (SPA) cathodes, as shown in Fig. 11 [134]. They observed that, for the gelatin-based cathode (Fig. 11a), the two distinct plateaus are present on the discharge profile even at a high current density of 1600 mA g^−1^, whereas for the PEO-based cathodes, the distinct plateaus disappear at current densities greater than 800 mA g^−1^ (Fig. 11b), which suggests two different discharge mechanisms for the two cells. XRD analysis of both cells pre- and post-discharge supported this hypothesis by revealing that in the gelatin-based cell, no elemental sulfur remained in the XRD spectrum, suggesting that all of the sulfur participated in the reaction which they accredited to the good dispersion properties of the gelatin-based composite conductive binding framework. Although the gelatin-based cathode retained both of the characteristic discharge plateaus at high current densities, the specific capacity for the entire discharge was only 29% of the expected theoretical capacity. The group postulated, as the first discharge region was relatively unchanged, that only a part of the dissolved long-chain PS was able to be fully reduced on the cathode surface due to already precipitated Li_2_S restricting ionic transport for the remaining active material. To counteract this phenomenon, a freeze-drying method was employed to increase the porosity in the gelatin-based framework to provide more reaction sites for complete PS reduction. SEM analysis revealed an increased porosity of the as-fabricated electrode with a corresponding specific capacity increased to 733 mAh g^−1^ when discharged at 1600 mA g^−1.^

Zhang et al. [164] further investigated the dispersion properties of gelatin-based host-free cathodes by controlling the pH of the aqueous electrode slurry to observe the influence on the homogeneity of the resultant electrode. The group found that the cathode prepared at pH 10 resulted in a more even dispersion of sulfur and conductive additives across the framework when compared to the cathodes prepared at pH 8 and pH 5. They hypothesized that the origin of this increase in dispersion in the framework was due to gelatin’s tendency to shift its conformation in solution when the pH is far from the isoelectric point (IEP). This increase in homogeneity resulted in a superior performance from the cathode fabricated at pH = 10, which delivered an initial discharge capacity of 1137 mAh g^−1^, compared with the 1024 mAh g^−1^ delivered by the pH 8 cathode and 1034 mAh g^−1^ delivered by the pH 5 cathode. The pH 10 cathode mediated a more complete redox reaction of the active sulfur, as evidenced by the strong re-emergence of the sulfur peak on the XRD spectrum after first discharge/charge.

**Fig. 12:**
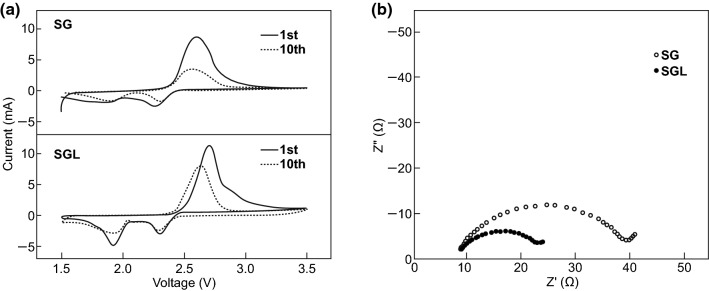
**a** CV of gelatin (SG) and l-cysteine gelatin (SGL) cathodes after 1st and 10th discharge with scan rate of 0.5 mV s^−1^ and **b** impedance plots for Li/S cells with SG and SGL after 1st discharge in the frequency range of (100 kHz–100 mHz). Reproduced with permission from Ref. [165]. Copyright 2012 Taylor and Francis

Jiang et al. [165] further improved the dispersion, adhesion, and electrochemistry of gelatin-based host-free cathodes through the introduction of l-cysteine onto the gelatin biopolymer framework. The incorporation of l-cysteine into this framework helped to reduce the polarization of the as-fabricated cathode, as evidenced by CV taken on the 1st and 10th cycle (Fig. 12a). EIS analysis also revealed a reduced charge-transfer resistance for the l-cysteine modified electrode (Fig. 12b), which the group attributed to the enhanced electronic network formed by the superior dispersion properties of the fabricated binding framework.

#### Sodium Alginate (SA)

The adhesion and dispersion properties of Na-alginate are well established; however, Bao et al. [135] found that polymeric frameworks based on Na-alginate can also initiate chemical interactions with the sulfur-active material. The group used the relative decrease in the obtained FTIR spectrum to confirm the interaction between the alginate and sulfur, which they postulate is the reason for the improved discharge capacity and capacity retention when compared with the sulfur cathode fabricated with PVDF.

#### Carboxymethyl Cellulose (CMC)

**Fig. 13:**
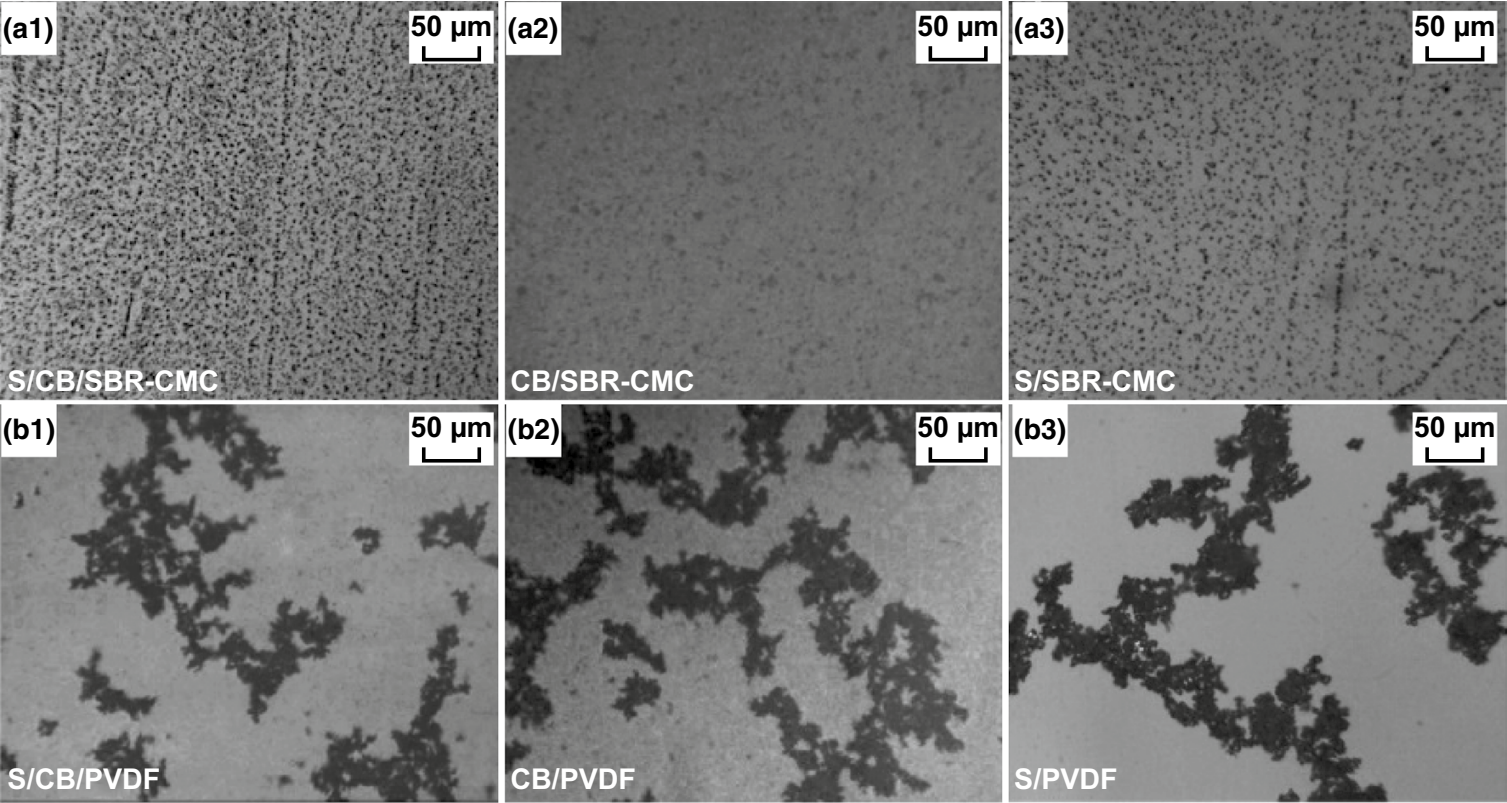
Dispersion morphology of **a1** S/CB/SBR–CMC, **a2** CB/SBR–CMC, **a3** S/SBR–CMC, **b1** S/CB/PVDF, **b2** CB/PVDF, and **b3** S/PVDF. Reproduced with permission from Ref. [136]. Copyright 2011 American Chemical Society

CMC, derived from cellulose, is a low-cost, water-soluble, and commercially available polysaccharide, which has found uses in medical applications, pharmaceuticals, cosmetics, and, most relevantly, as a thickener, dispersion aid, stabilizer, and binder in a verity of applications [166]. CMC can be used directly as a binder in the electrode manufacturing process but, due to its crystallinity, the electrodes fabricated in this matter are hard and rigid, and prone to cracking. As such, CMC is typically combined with styrene butadiene rubber (SBR) to increase the composites elasticity. The CMC/SBR blend is an attractive alternative to the conventional PVDF binder and is already finding commercial success in the manufacturing of graphite anodes for LIBs [23]. He et al. [136] applied a 1:1 blend of CMC/SBR to form a host-free PCF for Li–S batteries. The dispersion morphology of the electrode slurry was investigated via optical microscopy, as shown in Fig. 13. A clearly superior dispersion was obtained for the aqueous-based CMC/SBR (Fig. 13a1) slurry when compared with that of the PVDF-based slurry in NMP (Fig. 13b1). The group supposed that the addition of CMC into the slurry allowed the carbon black to be dispersed effectively as the carboxylate groups of the CMC can give rise to an effective surface charge on the carbon black, stabilizing the dispersion through an electrostatic double-layer repulsion effect. They further analyzed the dispersion properties by measuring the zeta potentials of the electrode components and verified the strong electrostatic repulsive force. Following this, the group suggested that this homogeneous dispersion of the CMC/SBR could result in a more effective conductive framework, which was supported by the low internal and charge-transfer resistance of the CMC/SBR-based composite determined by EIS analysis. As a result, the CMC/SBR-based cathode delivered a reversible capacity of 580 mAh g^−1^ after 60 cycles at 100 mA g^−1^ current density, far surpassing the reference electrode based on a PVDF binder.

#### Chitosan

**Fig. 14:**
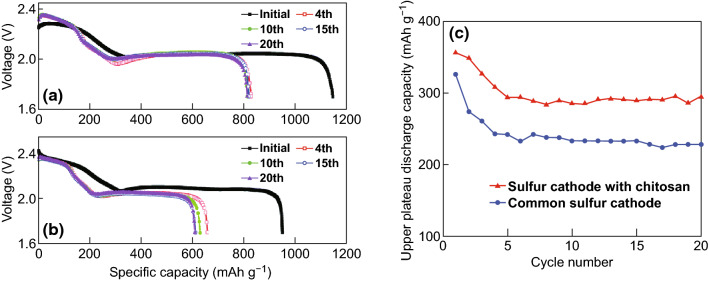
Discharge curves of batteries: **a** sulfur cathode with chitosan, **b** sulfur cathode with gelatin, and **c** the upper plateau discharge capacities of batteries with different sulfur cathodes. Reproduced with permission from Ref. [137]. Copyright 2015 Royal Society of Chemistry

Chitosan is an attractive natural polymer with a high nitrogen and hydroxyl content which is commonly sourced from crab and shrimp shells [167]. Chen et al. [137] applied chitosan as a chemical polysulfide anchor which was combined with acetylene black to form a host-free framework for Li–S batteries. Considerable improvements in the sulfur redox reversibility and cycling performance were achieved through the use of this binder, as evidenced by the higher reversible capacity displayed after cycling when compared with the gelatin-based cathode (Fig. 14). The authors attributed the higher upper plateau discharge capacities of the chitosan-based electrode to its polysulfide anchoring effect (Fig. 14c).

#### Gum Arabic (GA)

**Fig. 15:**
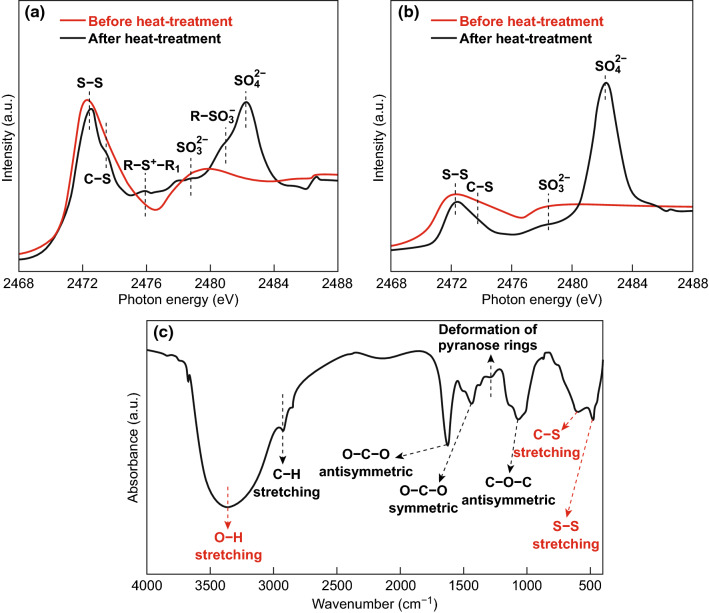
Characterization of chemical bonds between GA and S. **a** TEY XAS and **b** TFY XAS spectra for the mixture of GA and S before and after the thermal treatment at 80 °C. **c** FTIR spectroscopy of the mixture of GA and polysulfides (Li_2_S_*x*_, *x* = 8). Reproduced with permission from Ref. [138]. Copyright 2015 John Wiley and Sons

Gum Arabic (GA) is a tree gum exudate which has been utilized for over 5000 years in a variety of applications, including as an adhesive for paint and during the ancient Egyptian embalming process [168]. More recently, GA has been used as a thickening and stabilizing agent [169]. It is a branched, complex polysaccharide polymer, consisting of a main chain of β-d-galactopyranosyl, and side chains endowed with abundant carbonyl and nitrogen-containing functional groups [169]. Li et al. [138] adopted GA as a low-cost water-soluble binder to fabricate a host-free framework, wherein the GA allowed the electrode slurry to possess a good dispersion of active materials which resulted in a homogeneous electrode with reduced electrochemical impedance. The as-fabricated electrode delivered a high capacity of 841 mAh g^−1^ over 500 cycles at 0.5C with a high sulfur loading of 4.4 mg cm^−2^. Nanoindentation analysis revealed the GA displayed superior flexibility when compared with the gelatin and PVDF-based electrodes, which allowed for better delamination tolerance. X-ray absorption spectroscopy (XAS) was used to verify the chemical bonding between the GA and sulfur, while FTIR was used to verify bonding between GA and PS (Fig. 15). This analysis revealed that not only can the GA strongly hold sulfur through the host-free framework, it can also retain PS which can prevent migration and parasitic reactions at the lithium metal anode.

#### Guar Gum (GG)

Guar gum (GG) is yet another commonly used and widely available biopolymer [170]. GG can also be used to fabricate host-free frameworks for sulfur cathodes, as evidenced by Lu et al. [139]. When cycled at 1C, the resultant Li–S batteries delivered a reversible capacity of ≈ 600 mAh g^−1^ over 400 cycles. To explain the improved performance over the reference PVDF-based electrode, the group investigated the chemical and mechanical properties of the GG. FTIR analysis revealed the polar OH groups of the GG interact with both the sulfur and polysulfide species, which could have inhibited the shuttling effect, therefore increasing electrode stability and cell performance. The material’s behavior toward the electrolyte was observed for both GG and PVDF. It was found that the GG displayed limited swelling in the electrolyte, whereas PVDF was easily swollen, which the group supposed lead to the degradation of the bonding and conductive pathways in the PVDF framework. Furthermore, the GG sample displayed better viscosity (when measured in a 1 wt% solution) and hardness than the PVDF sample, all of which were suspected reasons for the superior performance of the GG framework in Li–S cells.

Following this, Cheng et al. [171] conducted a thorough investigation into how the rheological behavior of GG solutions changed with time, and the effects of these observations on the resultant dispersions of the electrode slurries. The group found that when an aqueous solution of GG was made, a gelatinous slurry (g-GG) was obtained; however after 48 h, the viscosity of the solution decreases and a better fluidity is obtained in a process called retrogradation (r-GG). FTIR spectroscopy was used to investigate this phenomenon, which revealed that the g-GG solution exhibited strong hydrogen bonding between the polymer and aqueous solvent; however, the r-GG solution preferred hydrogen bonding toward itself. Electrode slurries were constructed with both forms of GG and rheological analysis was conducted. The g-GG based slurry exhibited shear-thinning behavior, which suggested powder agglomerates were present; conversely, the r-GG slurry was more homogeneous. Consequently, electrodes fabricated from the r-GG slurry displayed better homogeneity and reduced agglomeration, as revealed by SEM analysis, which resulted in an increased electrochemical performance.

#### Carrageenan

**Fig. 16:**
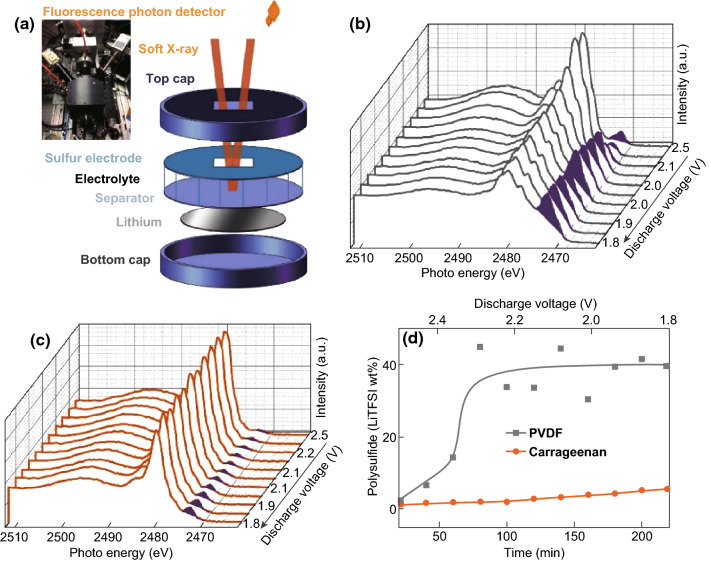
Operando XAS measurements of Li–S cell. **a** Schematic of the in situ XAS measurement set-up. The inlet photo is the actual customer build instrumentation for this experiment. **b**, **c** The S K-edge XAS spectra evolution of the electrolyte with voltage scan. The purple highlighted peaks are polysulfide adsorption peaks, which evolve during first discharge. PVDF binder-based Li–S cell shows the dramatic increase of polysulfide concentration in the electrolyte during the first lithiation process. The carrageenan binder-based Li–S cell shows much slow concentration built up of polysulfide. **d** The relative polysulfide concentration changes with discharge shows the superiority of carrageenan binder in immobilizing polysulfide. Reproduced with permission from Ref. [140]. Copyright 2017 Elsevier

Ling et al. [140] investigated a new method to achieve PS retention in Li–S batteries. By taking advantage of a nucleophilic substitution reaction between the polymer binder and polysulfides, a strong polysulfide anchoring effect was realized. By considering the reaction mechanism, the group determined that a sulfate group could serve as a suitable leaving group, therefore poly(vinyl sulfate) potassium salt (PVS) polymer was initially tried at a nucleophilic substitution binder for Li–S batteries. Although a strong PS anchoring effect was observed in the time lapse UV–Vis spectra and a C–S bond was formed between the binder and PS, the insufficient mechanical properties left room for improvement in terms of cycling stability. This led the group to investigate carrageenan as a nucleophilic substitution binder for Li–S batteries. Carrageenan is an aqueous-soluble natural product polymer with abundant sulfate groups and, furthermore, has a high amount of hydroxyl groups which provides enhanced adhesive capabilities. As with the PVS polymer, the carrageenan binder strongly adsorbed PS through the formation of a C–S bond, as determined by XAS and XPS analysis. The carrageenan binder allowed for a sulfur cathode with a high sulfur loading of 24.6 mg cm^−2^, which delivered an areal capacity of 33.7 mAh cm^−2^. The polysulfide retention was also demonstrated during cell operation using *operando* XAS measurements (Fig. 16). The cells were discharged at 0.2C between 2.6 and 1.8 V, while the fluorescence spectra were observed. As can be seen in Fig. 16b, the polysulfide concentration (purple peak) quickly increases and plateaus as the discharge proceeds; however, the polysulfide dissolution in the carrageenan-based electrode remains low (Fig. 16c), highlighting successful PS shuttling mitigation.

#### Starch

Starch is also a natural biopolymer also with good mechanical properties. Duan et al. [141] subjected starch to a gelatinization process before using the product to fabricate a host-free framework using only Super-P and commercial sulfur powder. The as-fabricated framework delivered a capacity retention of ≈ 90% at 0.2C after 200 cycles, which corresponds to a capacity decay of only 0.05% per cycle. SEM analysis revealed that before cycling, the starch-based framework displayed quite a severe degree of agglomeration, which the authors ascribed to the greater wettability of Super-P and sulfur particles compared with the starch, however after 100 cycles the integrity of the framework remained intact. Conversely, the PVDF framework displayed cracks throughout the composite, resulting in electronically isolated sections of electrode. The authors attributed this stability to the minimal swelling of the gelatinized starch framework when the framework was exposed to the electrolyte. This resistance to swelling allowed the structure to avoid the exfoliation of sections of the electrode. In short, natural polymers often display the necessary viscosity in solutions to form suitable binders and are often naturally endowed with specialized functional groups conducive to good host-free sulfur cathode function.

Overall, natural polymers have many inherent benefits. The aqueous-soluble and cheap natural polymers could be combined with sulfur hosts which are synthesized through cheap and green chemical methods to reduce the environmental impact of Li–S cell fabrication while still obtaining a high electrochemical performance. A relatively small amount of research has been carried out with multifunctional sulfur hosts combined with natural polymers, which may be a fruitful future research direction.

### Synthetic Polymers

The wide range of available synthetic polymers have the advantage of being highly tailorable so that favorable mechanical properties and a strong binding force in host-free PCFs can be achieved.

#### Poly(Vinylpyrrolidone) (PVP)

**Fig. 17:**
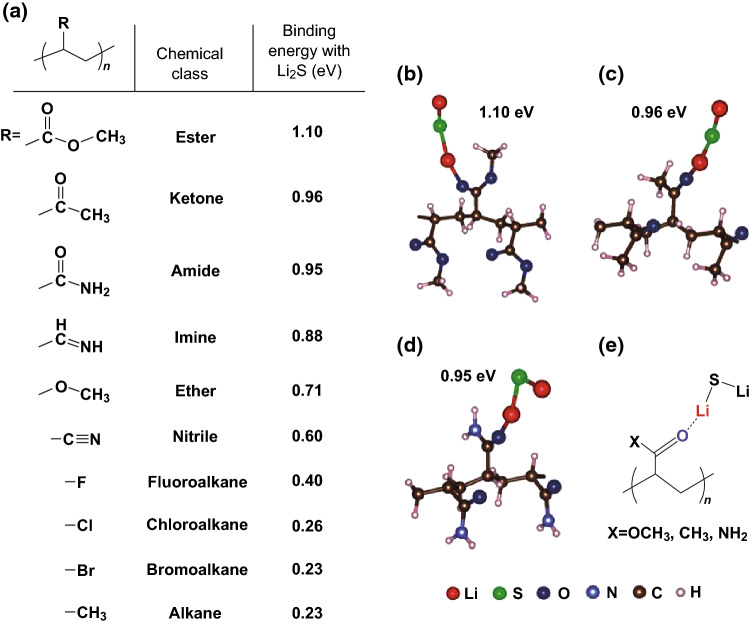
**a** Table showing the calculated binding energy of Li_2_S with various functional groups (*R*) based on the framework of vinyl polymers –(CH_2_–CHR)_*n*–_. **b**–**d** Ab initio simulations showing the most stable configuration and calculated binding energy of Li_2_S with **b** ester, **c** ketone and **d** amide R groups in vinyl polymers. **e** General schematic representing the Li–O interaction between Li_2_S and >C=O groups as shown in **b**–**d**. Reproduced with permission from Ref. [142]. Copyright 2013 Royal Society of Chemistry

The crucial work by Seh et al. [142] provided the theoretical insight into how to achieve a strong chemical bonding mechanism between binders and polysulfides. The group used Ab initio simulations in the framework of density functional theory (DFT) to evaluate the interactions between various functional groups (*R*) and Li_2_S on a vinyl polymer [–(CH_2_CHR)_*n*_–] framework (Fig. 17). They found that a lithium atom in Li_2_S is capable of forming coordination-like bonds with electron-rich groups containing lone pairs of electrons on oxygen, nitrogen, halogens, etc. The strongest interaction was determined to be between Li_2_S and carbonyl (>C=O) groups, found in esters, ketones, and amides, as shown in Fig. 17a. The group attributed this strong binding to the hard-acid properties of Li^+^, which can interact with the hard oxygen donor atoms in the carbonyl groups to form a strong lithium-oxygen bond (Li–O). Conversely, the interaction between fluoroalkane groups and Li_2_S are much weaker, which provides insight as to why the PVDF binder cannot act as a polysulfide anchor. Considering this, the group selected PVP to act as a multifunctional binder to construct a polymeric framework using Li_2_S as an active material. Evidence of the strong interaction between the active material and the binder was provided by observing the high degree of dispersion in the electrode slurry, which the authors attributed to the strong adsorption of PVP onto the Li_2_S particles, which stabilized the dispersion. Upon cycling at 0.2C, the as-fabricated batteries retained 69% of their original capacity, corresponding to a low 0.062% capacity loss per cycle attributed to a strong PS retention effect. The group quantified the PS anchoring effect by conducting inductively-coupled plasma-optical emission spectroscopy (ICP-OES) analysis on the electrolyte after discharge, which showed that the PVP-based electrode showed consistently reduced amounts of sulfur in the electrolyte after 1, 5, 10, and 20 cycles.

An interesting phenomenon regarding the solvent effects on slurry dispersions was investigated by Fu et al. [172]. The group found that by using acetic acid (AA) as a co-solvent, the dispersion properties of aqueous PVP and PAA slurries was substantially increased due to a chain opening effect. The co-solvent based slurries displayed an increased viscosity, with a correspondingly enhanced porosity, uniformity, and mechanical properties of the electrodes fabricated by this approach. The PVP-based host-free cathode fabricated using the AA co-solvent approach delivered an initial discharge capacity improvement of 220 mAh g^−1^ over the PVP-based framework cast from a pure water slurry. The long-term cycling performance of the AA co-solvent approach was also improved, delivering a reversible capacity of 530 mAh g^−1^ after 100 cycles at 0.3 A g^−1^.

#### Poly(Ethylene Oxide) (PEO)

**Fig. 18:**
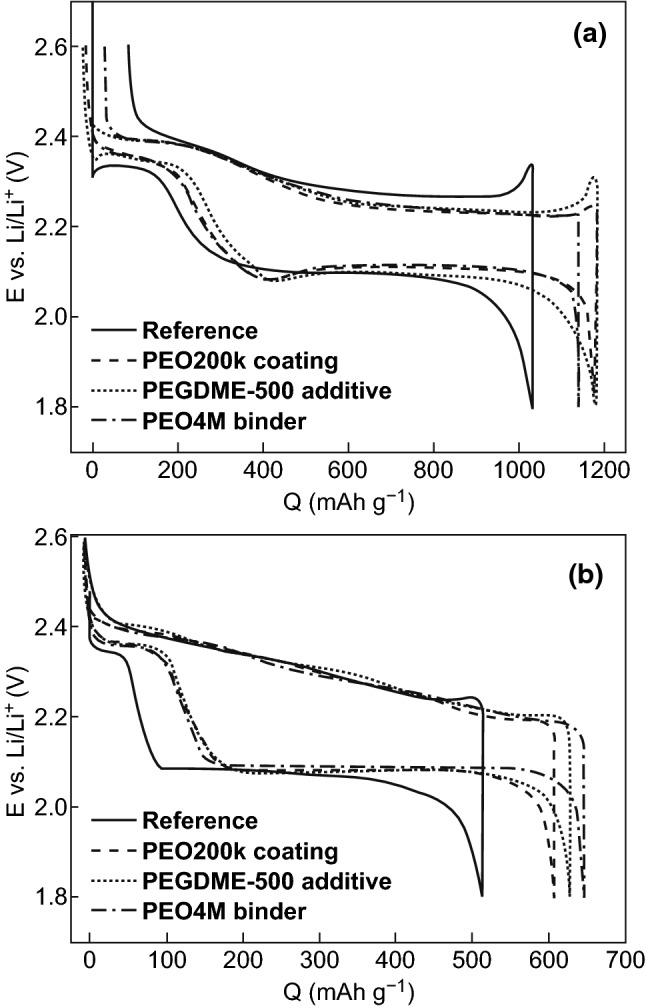
Voltage profiles for the reference and PEG/PEO-modified cells at C/5 for **a** the 1st cycle and **b** 50th cycle. Reproduced with permission from Ref. [143]. Copyright 2013 Royal Society of Chemistry

One of the primary considerations when fabricating host-free PCFs with traditional binders, which swell or dissolve in the organic electrolyte, is that the void structure required to house the sulfur is lost during sulfur dissolution. As such, the interaction between the binder and the electrolyte in host-free cathodes is an important consideration. This can be elucidated through the investigation of PEO as a binder in host-free frameworks [173, 174]. Lacey et al. [143] investigated the mechanisms by which PEO binders can improve sulfur cathode performance. As lower molecular weight polymers (i.e., PEG-20000) are soluble, and higher molecular weight PEO (*M*_w_ ≤ 4,000,000) swell in common liquid electrolytes, they considered it unlikely that PEO coatings can physically retard dissolved polysulfides during cycling. Upon observing the voltage profile for the first cycle (Fig. 18a), they observed that the voltage peak at the beginning of the charge cycle, which has been attributed to cell polarization due to insoluble discharge product deposition, is removed entirely when PEO is used as a binder. After 50 cycles, the PEO-based composite conductive framework enables a high capacity retention with distinct upper and lower voltage plateaus during discharge (Fig. 18b). They concluded that the PEO binder enabled an improvement in electrochemical reversibility and a suppression of passivation on the sulfur cathode due to the nature of PEO dissolution (or swelling) which modified the electrolyte system.

**Fig. 19:**
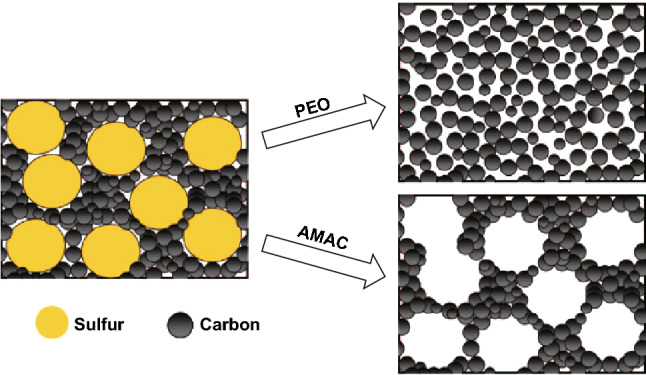
Schematic structure of the sulfur cathode before and after PS dissolution. Reproduced with permission from Ref. [144]. Copyright 2012 The Electrochemical Society

Further investigation into the swelling/dissolution phenomenon of PEO binders was conducted by Zhang [144], who argued that polymeric frameworks which are based on polymer binders that dissolve or swell in common liquid electrolytes cannot maintain a stable void structure during sulfur dissolution and are thus unsuitable for Li–S cells with a long cycle life (Fig. 19).

#### Poly(Acrylamide-*co*-Diallyldimethylammonium Chloride) (AMAC)

In response to this swelling/dissolution phenomenon observed with PEO binders, S.S. Zhang introduced a cationic polyelectrolyte named poly(acrylamide-*co*-diallyldimethylammonium chloride) (AMAC) which is substantially insoluble in organic electrolytes but highly soluble in water [144].

**Fig. 20:**
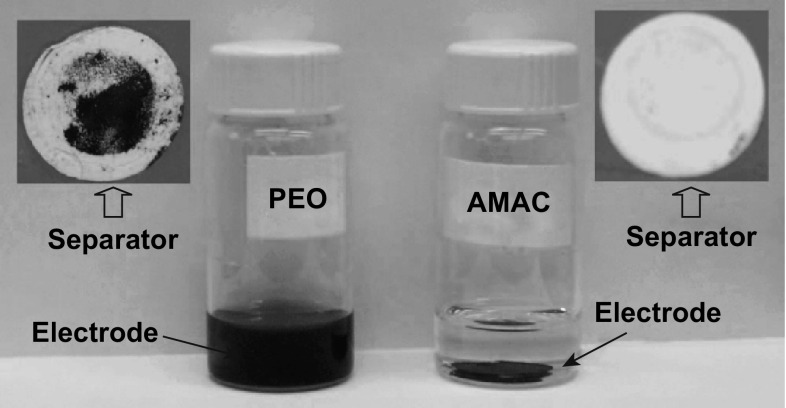
Visual pictures of separator and sulfur cathode after the Li/S cell was discharged to 300 mAh g^−1^ sulfur and the cathode was stored in triglyme at 60 °C for 48 h. Reproduced with permission from Ref. [144]. Copyright 2012 The Electrochemical Society

The author attributed the enlarged second discharge plateau to the retained pore structure after sulfur dissolution which allowed for easy deposition of Li_2_S_2_ and Li_2_S. To further illustrate this effect, the group partially discharged the PEO- and AMAC-based cathodes to 300 mAh g^−1^ to achieve a total conversion of solid sulfur to soluble PS in order to observe the behavior of the composite conductive binding framework after PS dissolution. The partially discharged cells were disassembled and washed with electrolyte before being stored in triglyme. Due to the gelation of the PEO-based cathode, many of the electrode components were stuck to the separator after disassembly, in contrast to the AMAC-based cathode which maintained its structural integrity after disassembly, as shown in Fig. 20. Furthermore, the AMAC-based cathode could withstand storage in the triglyme solvent for 48 h at 60 °C, which was not the case for the PEO-based electrode. These results revealed that the AMAC-based composite conductive binding framework delivers a greater structural integrity and void structure compared with PEO-based composites. Electrochemical results reinforced this claim, with the AMAC-based cathode delivering a reversible capacity of 652 mAh g^−1^ after 100 cycles compared with 384 mAh g^−1^ for the PEO-based cathode. This work highlights the importance of the interaction between the electrolyte and binder, especially during the fabrication of host-free sulfur cathodes.

#### Poly(Acrylic Acid) (PAA)

**Fig. 21:**
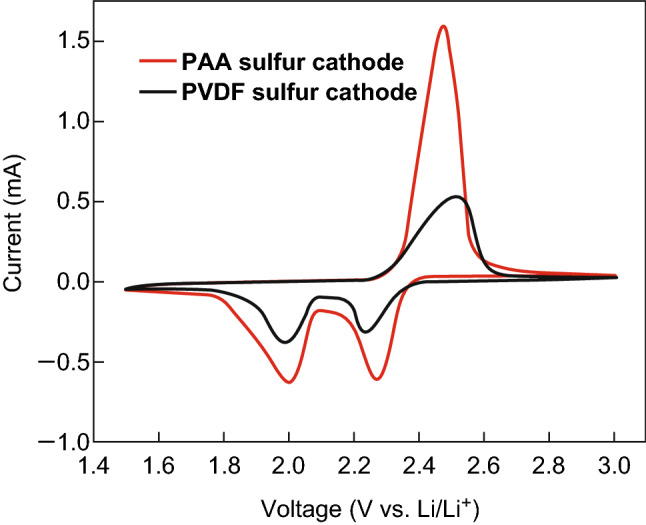
Cyclic voltammogram curves of the Li–S cells with PAA sulfur cathode and PVDF sulfur cathode at a scan rate of 0.1 mV s^−1^. Reproduced with permission from Ref. [145]. Copyright 2012 The Electrochemical Society

PAA, a mechanically robust, water-soluble polymer was first investigated by Zhang et al. [145] as a polymeric binder in Li–S batteries. Not only did the PAA-based cathode delivered a higher discharge capacity than the PVDF-based cell, it also displayed an almost twofold increase in the reduction current and threefold increase in the oxidation current when observing the CV (Fig. 21), which suggests better reaction kinetics within the PAA electrode. The group suggested the strong binding strength helped to stabilize the electrode framework, restrain polysulfides, and prevent delamination of the electrode.

#### LA132

**Fig. 22:**
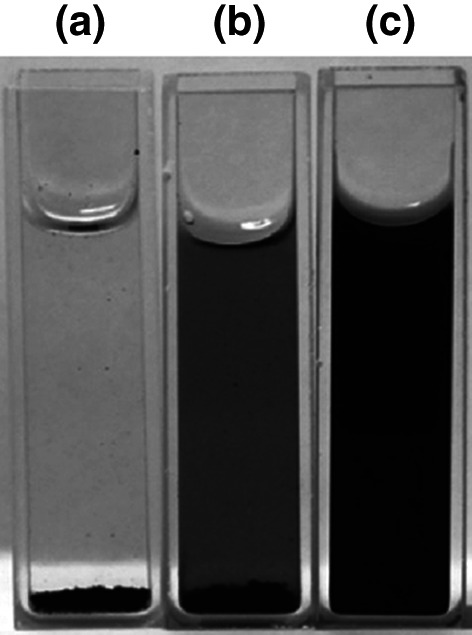
Suspensions of 1 mg mL^−1^ sulfur composites dispersed in **a** SA; **b** CMC; **c** LA132 after an overnight settlement. Reproduced with permission from Ref. [175]. Copyright 2016 Elsevier

LA132, a flexible, water-soluble, highly adhesive copolymer containing acrylonitrile, acrylate, and acrylamide, was investigated as a binder in Li–S batteries by Hong et al. [175]. The dispersions of electrode slurries using SA, CMC, and LA132 were compared (Fig. 22), and it was found that the LA132-based slurry provided the best dispersion after being left overnight. This corresponded to a more homogenous and stable cathode, which allowed for a higher discharge capacity over 50 cycles. Pan et al. [146] demonstrated that a cathode fabricated with a 5 wt% loading of LA132 binder could even outperform a cathode using 10 wt% of PVDF.

#### Poly(Amidoamine) (PAMAM)

Poly(amidoamine) (PAMAM) is a highly branched synthetic polymer referred to as a dendrimer, which possess a central core, repeating interior branch cells, and terminal functional groups [176]. Bhattacharya et al. [147] compared various PAMAM dendrimers with different functional groups as aqueous-soluble binders for Li–S batteries. Most importantly, the cathodes based on PAMAM dendrimers with hydroxyl (G4OH), 4-carboxymethylpyrrolidone (G4CMP), and carboxylate (G4COONa) functionality enabled a high sulfur loading above 4 mg cm^−2^, comparatively greater than the reference CMC/SBR-based cathodes (2.34 mg cm^−2^). As predicted by Seh et al.’s work [142], the previously mentioned PAMAM dendrimers with carbonyl functional groups enabled chemical anchoring of PSs within the cathode framework, as evidenced by XPS analysis. Not only that the dendrimers also display an internal porous structure in the range of 2 nm, which could physically trap PS, resulting in a dual-approach PS restriction at the cathode. All things considered, the PAMAM dendrimer framework enabled a high sulfur loading in the composite (> 68 wt%), a high areal capacity (4.32 mAh cm^−2^), and a capacity retention of ≈ 640 mAh g^−1^ after 100 cycles.

#### Poly(Ethylenimine) (PEI)

Poly(ethylenimine) is an amine containing polymer which has been used as a chemical PS trap in Li–S batteries [177]. Zhang et al. [148] used PEI as both a binder and PS anchor to form a host-free framework. A high sulfur loading of 8.6 mg cm^−2^ was achieved throughout the composite, which delivered a reversible capacity of 744.2 mAh g^−1^ after 50 cycles. UV–Vis and XAS analysis revealed direct evidence of electrostatic interaction between the amino groups in the PEI with PS intermediates, resulting in a reduction in PS shuttling and a subsequent improvement in the electrochemical performance. Wang et al. [178] subsequently modified PEI polymer with methyl iodide (CH_3_I), which resulted in an even greater PS anchoring ability; thus, a further improved electrochemical performance was obtained.

#### Polycationic Binders

**Fig. 23:**
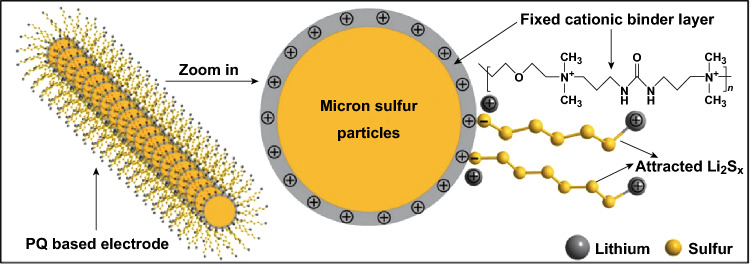
Polysulfides confinement through cationic polymer. The electrostatic attraction between the PQ quaternary ammonium cations and polysulfide anions. Reproduced with permission from Ref. [149]. Copyright 2017 American Chemical Society

An electrostatic confinement of polysulfide intermediates was realized through the use of a cationic polymer binder by Ling et al. [149]. The poly[bis(2-chloroethyl) ether-alt-1,3-bis[3(dimethylamino) propyl]urea] quaternized (PQ) binder, endowed with quaternary ammonium cations (Fig. 23), binds with the soluble polysulfide (Li_2_S_6_) with an energy of 1.89 eV, but is lower than the covalent bonding energy thus providing an electrostatic interaction. The retention abilities of the PQ binder were evaluated experimentally through time lapse UV–Vis spectroscopy, which found that the PQ binder is able to reduce the amount of polysulfides in the solution through electrostatic interaction, whereas the concentration in the PVDF experiment remains unchanged. Electrochemical characterization revealed the PQ displayed good separation of discharge plateaus and delivered a high areal capacity of 9 mAh cm^−2^ with a sulfur loading of 7.5 mg cm^−2^.

**Fig. 24:**
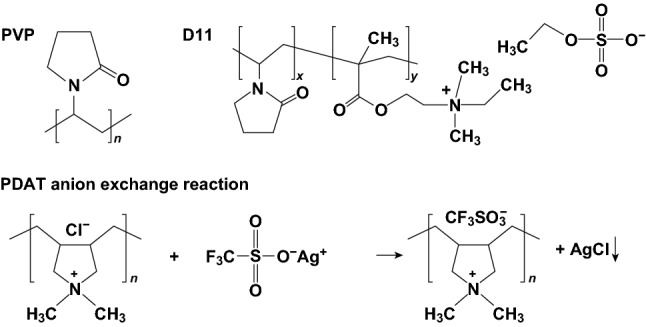
Molecular structures of polyvinylpyrrolidone (PVP) and poly[(2-ethyldimethylammonioethyl methacrylate ethyl sulfate)-*co*-(1-vinylpyrrolidone)] (D11) and the SYNTHESIS of poly(diallyldimethylammonium triflate) (PDAT) via anion exchange. Reproduced with permission from Ref. [125]. Copyright 2017 American Chemical Society

Two representative cationic binders for Li–S batteries were investigated by Su et al. [125]. The group used poly[(2-ethyldimethylammonioethyl methacrylate ethyl sulfate)-*co*-(1-vinylpyrrolidone)] (D11) and poly(diallyldimethylammonium triflate) (PDAT), synthesized through an anion exchange reaction between poly(diallyldimethyl ammonium chloride) and silver triflate (CF_3_SO_3_Ag) as shown in Fig. 24, and compared the electrochemical performance with a PVP-based Li–S cell. D11 and PDAT were chosen due to their positively charged nitrogen atom, while PVP contains an uncharged nitrogen, so that the role of a positively charged nitrogen in PS anchoring could be investigated. Both the D11- and PDAT-based Li–S cells delivered an improved cycling performance compared with the PVP-based cell. Although the D11-based cell delivered a similar initial discharge capacity compared to the PVP, its capacity retention over 50 cycles was improved. The PDAT binder-based electrode delivered both an increased initial discharge capacity as well as an improved capacity retention over both the PVP- and D11-based electrodes; thus, the PDAT binder was further examined. Short duration polysulfide adsorption tests (i.e., <1 min) revealed the PDAT binder composite displays a superior anchoring effect, which was confirmed by UV–Vis spectroscopy. Furthermore, XPS analysis of the lithiated electrodes revealed a superior sulfur utilization for the PDAT-based electrode. The group concluded that polycation containing binders can mediate a stronger PS sequestration.

Liao et al. [179] investigated the effect of the chosen counter anions on poly(diallyldimethylammonium) (PDADMA)-based binders for Li–S batteries. The chosen counter anions in this case were: Cl^−^, PF_6_^−^, BF_4_^−^, and TFSI^−^. The group found that the PDADMA with the latter 3 counter anions could successfully anchor PS, whereas the PDADMA with a Cl^−^ counter anion was ineffective at PS trapping. The TFSI^−^ anion based binder delivered the lowest capacity decay and lowest polarization while maintaining the best cycling stability.

#### Polymeric Ionic Liquids (PILs) Binders

**Fig. 25:**
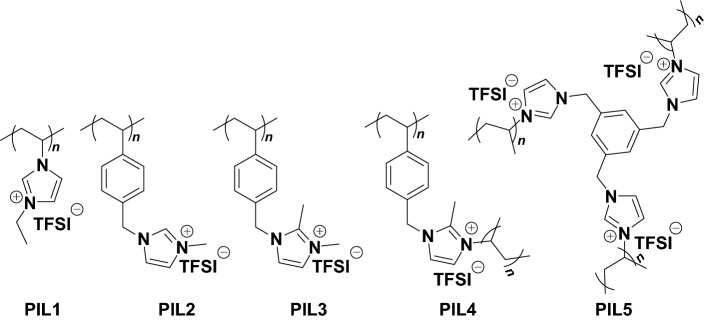
PIL binders examined for use in Li–S systems. Reproduced with permission from Ref. [150]. Copyright 2018 American Chemical Society

Five different polymer ionic liquids (PILs) were investigated as cathode binders in Li–S batteries by Vizintin et al. [150]. Of particular interest was PIL4 (Fig. 25), which enabled a discharge capacity of 1015 mAh g^−1^ after 3 cycles, 657 mAh g^−1^ after 200 cycles, and 446 mAh g^−1^ after 500 cycles. The group found that between the 50th and the 200th cycle, the ratio between the capacity of the upper voltage discharge plateau (*Q*_high_) and the total discharge capacity (*Q*_total_) increased, which they related to a more efficient reduction of sulfur to Li_2_S_4_ during prolonged discharge-charge cycling. To obtain possible reasons for the increased cycling performance, post-mortem SEM analysis was carried out. The PVDF and PIL electrodes in the discharged state displayed a different morphology, which the authors suggested was due to an increased uptake of ionic compounds by the PIL binder, thus mediating a more uniform mixing and retention of sulfide species within the PIL binding framework. This was supported by submerging the PIL4 in a PS solution, which noticeably swelled and formed a white gel. Overall, the authors attributed the increased cycling performance of the PIL4 by its ability to provide sufficient adhesion, improve sulfur redox and dispersion, and trap polysulfide during swelling/deswelling cycles thus reducing volume change-induced stress throughout the framework.

#### Thiokol

Thiokol, a type of synthetic polysulfide rubber, was also adopted as functional binder for restricted polysulfide shuttling in Li–S batteries by Liu et al. [151]. The group proposed that the Thiokol could act as a kind of polysulfide scissor which could reduce the amount of long-chain PS, thereby reducing the PS shuttle. A similar mechanism was also reported when dithiothreitol was used as an electrolyte additive [180]. The thiokol-based binder is insoluble in the electrolyte which, as mentioned earlier, results in a stable structure during cycling [144]. As a result, the thiokol-based cathode delivered an initial discharge capacity of 819 mAh g^−1^ at 0.1C and achieved a capacity retention of 61.1% after 200 cycles.

#### Ammonium Polyphosphate (APP)

**Fig. 26:**
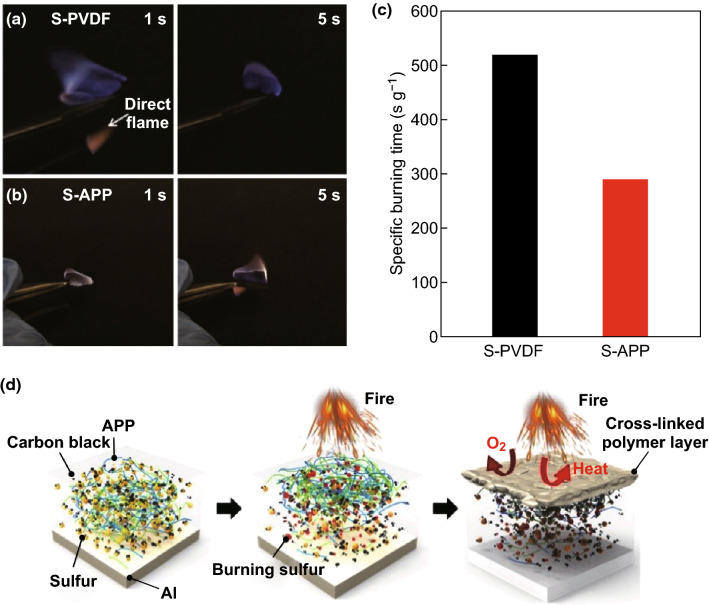
Flame-retardant properties. The specific burning time test of sulfur electrodes with **a** S-PVDF electrode and **b** S-APP electrode. The times indicated in the pictures are counted as soon as the electrodes are exposed to the direct flame from a lighter (indicated by the white arrow in panel **a**). **c** The specific burning time of the sulfur cathodes with APP and PVDF binders. **d** Schematic showing the flame-retardant mechanism of the APP binder-based sulfur electrode. Reproduced with permission from Ref. [77]. Copyright 2018 American Chemical Society

The binder materials reviewed thus far have been based on organic polymer backbones; however, the work by Zhou et al. [77] demonstrates this does not necessarily have to be the case. The group used the inorganic polymer ammonium polyphosphate (APP), a commercially available food additive, emulsifier, and fertilizer as a multifunctional binder in Li–S cathodes. In contrast to traditional polymers based on an organic C–C backbone (which cannot mediate PS anchoring), the backbone of the APP polymer can indeed initiate polysulfide trapping due to its polar nature. Evidence of the PS retention by the APP binder was provided by way of adsorption experiments and UV–Vis spectroscopy, which revealed a strong decrease in polysulfide concentration when exposed to APP polymer. DFT calculations were conducted using a range of polysulfide species (Li_2_S_*x*_ where *x* = 1, 2, 4, and 8), which showed that the APP binder delivered binding energies in the range of 2.16 to 2.30 eV, much higher than the PVDF binder can achieve (0.58 to 0.74 eV). Further evidence for the APPs superior polysulfide retention was given by the stability of the open circuit voltage (OCV) over a 30-day period. The voltage of the APP binder-based Li–S cell showed almost no decrease in OCV, whereas the PVDF-based cell’s voltage dropped from 2.42 to 2.29 V, suggesting the reduction of sulfur to PS had begun. When electrochemical testing was carried out, the APP binder allowed for an active material loading of 5.6 mg cm^−2^ to be achieved, which delivered a reversible discharge capacity of 530 mAh g^−1^ after 200 cycles at 0.5C. The group also carried out burning time tests which showed the flame-retardant properties of the APP binder could increase the safety of Li–S cells, as shown in Fig. 26.

In summary, synthetic polymers have been remarkably successful in improving the mechanical properties, sulfur loading, and PS anchoring abilities in PCFs. Future works with synthetic polymers could aim to improve the E/S ratio of Li–S cells as well as improve safety through the inclusion of flame-retardant materials.

### Composite Binders

Composite binders can be synthesized by combining two different polymers, which may result in a synergistic performance which is greater than the sum of their parts. For example, Lacey et al. [152] investigated a combination of PVP and PEO as a binder for Li–S cathodes. The group found that a 1:4 mixture of PVP:PEO delivered the highest capacity after 50 cycles at 0.2C, outperforming both pure PEO- and pure PVP-based electrodes as well as a 2:3 CMC/SBR-based electrode.

Jung et al. [153] utilized a small amount of poly(ethyleneimine) (PEI) to form a PVP-based composite binder. The PEI functions as a cationic dispersant which can stabilize aqueous dispersions as well as increase the adhesion of paints, inks, and pigments on different surfaces. The work showed that by increasing the PEI loading in the electrode slurry from 0.25 to 2.5%, the resultant viscosity of the 5% PVP solution increased from 14 to 120 cP, providing a suitable slurry for electrode coating while also increasing the frameworks stability in the electrolyte. These characteristics allowed the PVP/PEI composite binder to deliver a higher electrochemical performance compared with a framework based on PVP alone.

Ahktar et al. [154] fabricated a composite binder (GPC) by combining PEI and gelatin. Gelatin was chosen due to its established dispersion and adhesion properties, and PEI was utilized for its PS trapping ability. Interestingly, when subjected a PS solution, the GPC binder displayed better PS trapping than either of the individual components of the composite, as verified by UV–Vis spectroscopy. Owing to the adhesion, strong dispersion, and PS anchoring, the GPC based electrode delivered a reversible capacity of 871.3 mAh g^−1^ at 0.2C after 100 cycles.

Kim et al. [181] investigated the effect different binders had on the resultant porosity of Li–S cathodes. The group fabricated composite binders using CMC:PTFE, PVA:PTFE, and various *M*_w_ PVP before conducting BET measurements on the product. The average pore diameters in different cathodes were found to be around 20 and 0.05 µm, regardless of the type of binder; however, the electrodes with PTFE binders displayed an increased specific surface area (SSA). Upon electrochemical investigation, the group found that the PTFE:CMC based framework delivered a higher operating voltage and a sulfur utilization approaching 70%, which they suspected was due to the lower interfacial resistance in accordance with the increased surface area.

### Cross-Linked Binders

**Fig. 27:**
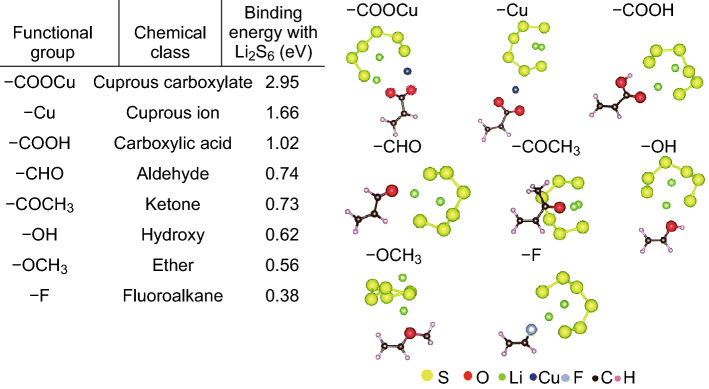
Binding energies of Li_2_S_6_ with various functional groups. Reproduced with permission from Ref. [155]. Copyright 2018 Royal Society of Chemistry

Further improvements into the mechanical properties of binders can be realized through a cross-linking mechanism. Liu et al. [155] fabricated a robust network binder through a ionic cross-linking effect using SA and Cu^2+^ ions. As mentioned previously, the oxygen rich groups on polymeric binders can mediate a coordination like interaction toward the Li^+^ ions in polysulfides, however this work found that a more efficient PS anchoring effect can be realized through direct interaction between the polysulfide anions and cations in the polymer binder (i.e., Cu^2+^). As evidenced by DFT calculations, the strongest polysulfide constraint are obtained when a synergistic electronegative and electropositive anchoring is achieved (Fig. 27). Adsorption tests along with UV–Vis spectroscopy confirmed the strong anchoring effect of the SA/Cu^2+^ binder, which corresponded to an increase in electrochemical performance. The Li–S cell based on the SA/Cu^2+^ framework delivered an 83% capacity retention over 100 cycles, a discharge capacity of 758 mAh g^−1^ after 250 cycles at 1C, and when the rate performance was evaluated, the cell delivered an outstanding capacity of 586 mAh g^−1^ at 6C. This work shows the electropositive/electronegative approach toward polysulfide anchoring can show favorable retention.

**Fig. 28:**
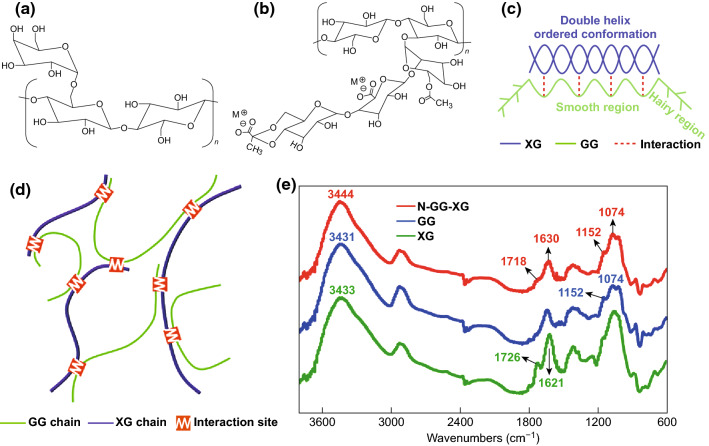
Chemical structures of **a** GG and **b** XG; **c** schematic of the intermolecular binding effect between GG and XG; **d** schematic of the polymer network formed by the intermolecular binding effect; and **e** FTIR spectra of GG, XG, and N-GG-XG. Reproduced with permission from Ref. [156]. Copyright 2017 Royal Society of Chemistry

A mechanically robust composite guar gum and xanthan gum (XG) binder was developed by Liu et al. [156], as shown in Fig. 28. Xanthan gum, similar to guar gum, is a natural polysaccharide biopolymer; however, the side chains in the XG polymer contain acetic and pyruvic acid residues (Fig. 28b) [182]. Hydrogen bonding occurs between the XG polymer and “smooth” regions (i.e., areas along the polymer backbone with no galactose residues) of the GG polymer, and as a result a mechanically robust biopolymer network is synthesized (Fig. 28c). The group examined the intermolecular interactions of the network through FTIR spectroscopy (Fig. 28e), which indicated the interaction had occurred, with the optimal ratio between the GG and XG determined to be 3:1 (GG:XG). The mechanically robust binding framework, with abundant functional groups from both polymers, allowed for an ultra-high sulfur loading of 19.8 mg cm^−2^ to be achieved, which delivered an areal capacity of 26.4 mAh cm^−2^.

**Fig. 29:**
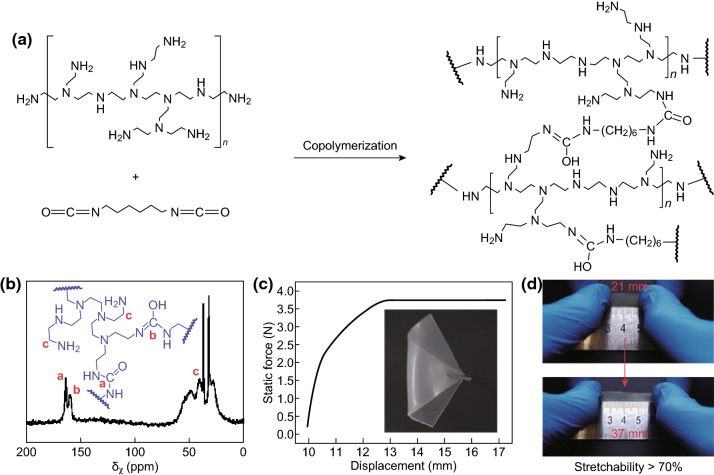
**a** Synthesis scheme of AFG binder by copolymerization of PEI with HDI in DMF solution. **b** ^13^C NMR spectrum of AFG, and the resonance amide signal at 163.58 ppm (red **a**, N–CH=O) with 159.45 ppm (red **b**, N=C–OH) and amine group signal at 40.09 ppm (red **c**, C–C–NH_2_) were detected. **c** Tensile property test of the AFG binder, which reveals the flexibility of the AFG binder polymer. **d** Digital photographs show the AFG copolymer with excellent stretchability. Reproduced with permission from Ref. [157]. Copyright 2017 John Wiley and Sons. (Color figure online)

Chen et al. [157] created a 3D hyperbranched polymer network through the copolymerization of PEI and hexamethylene diisocyanate (HDI) to form the amino functional group (AFG) binder (Fig. 29). The covalent bonding between the PEI and HDI was verified though ^13^C NMR spectroscopy and XPS analysis. This covalent network enabled the AFG binder to be stretched >70% without damage (Fig. 29d). When electrodes were fabricated using the AFG binder, they delivered a 91.3% capacity retention over 600 cycles at 2C. Following this, the group conducted a series of experiments to explain the low capacity fading. In situ UV–vis spectroscopy was used qualitatively monitor the discharge products and found that the polysulfides were released from the PVDF-based electrode far faster than from the AFG-based cell. DFT analysis was also carried out, which revealed considerable binding between the amino groups in the AFG backbone.

Although it possessed interesting physical and electrochemical properties, the AFG binder was insoluble in common solvents used in electrode slurries. This prompted the Chen et al. [158] to further develop a cross-linked PEI and poly(ethylene glycol) diglycidyl ether (PEGDGE) composite binder, named PPA, which was hydrophilic and thus water soluble. The PPA binder displayed excellent adhesion strength and was strong enough to support up to a 100 g weight, unlike PVDF, which was unable to support any weight. As a result of its excellent adhesion and chemical polysulfide anchoring, the Li–S batteries based on this cross-linked binder delivered outstanding electrochemical performances.

Yan et al. [159] introduced a robust network structure based on PEI and epoxy resin (ER). The group tailored the ratio between the framework components and found that a ratio of PEI:ER between 1:1 and 1:4 delivered a binder with unnoticeable deformation toward the electrolyte after 7 days submersion. UV–Vis spectroscopy was carried out to observe the binder’s polysulfide anchoring ability, with the peak relating to polysulfides approaching zero. The mechanical properties of the PEI/ER binder with different component ratios were also examined. A 1:1 ratio delivered a relatively low tensile strength of 1.5 MPa, which the author’s attributed to insufficient cross-linking, however when the ratio was increased to 1:2 a tensile strength of 22.3 MPa was obtained, which increased to 27.5 and 29.6 MPa for 1:3 and 1:4 based composites, respectively. Electrochemical testing revealed the PEI/ER_1:2_ binder delivered a discharge capacity of 829 mAh g^−1^ after 1000 cycles at 0.5C, which was increased to 937 mAh g^−1^ after 1000 cycles with the inclusion of a PEI/ER/Super-P interlayer.

Composite and cross-linked binders excel when two or more outstanding properties of individual materials are synergistically utilized. Further improvements in the overall performance, loadings, electrolyte content, and safety of Li–S cells could be realized through the rational combination of composite/cross-linked binders and a relevant sulfur host.

## Multifunctional Polymer Composite Frameworks

The research reviewed thus far typically utilizes polymeric binders to form robust networks which can retain the sulfur and electrode components. However, multifunctional binders can fulfil more than one role in the composite. For example, electronically conductive binders can fill the role of both binder and conductive additive. Though some of the papers in this section mention multifunctional polymers in host@PCFs, the research into multifunctional binders in host-free PCFs is emphasized in this section. A table containing the electrochemical performances of multifunctional PCFs is included in Table 4.

**Table 4:** Binders and their electrochemical performance in multifunctional PCFs

Binder	Discharge capacity @ *n*th cycle	C-rate	References
*Electronically conductive binders*			
PAA/PEDOT:PSS	833 mAh g^−1^ @ 80 cycles	0.5C	[183]
PEDOT:PSS/Mg^2+^	≈ 810 mAh g^−1^ @ 250 cycles	0.5C	[184]
Polyaniline (PANi)	439 mAh g^−1^ @ 50 cycles	≈ 0.07C	[185]
Polypyrrole (PPy)/polyurethane	≈ 1000 mAh g^−1^ @ 100 cycles	0.33C	[186]
Poly(9,9-dioctylfluorene-*co*-fluorenone-*co*-methylbenzoic ester)	≈ 800 mAh g^−1^ @ 150 cycles	0.1C	[187]
*Ionically conductive binders*			
Li-Nafion	≈ 540 mAh g^−1^ @ 100 cycles	0.2C	[115]
Li-Nafion/PVP/nano silica	≈ 800 mAh g^−1^ @ 350 cycles	1C	[188]
Sulfonated poly (ether ether ketone)	≈ 300 mAh g^−1^ @ 300 cycles	≈ 0.6C	[189]
PEO/tannic acid	476.7 mAh g^−1^ @ 1000 cycles	0.2C	[190]
*Redox-active binders*			
*π*-Stacked perylene bisimide	600 mAh g^−1^ @ 150 cycles	1C	[191]
Naphthalene–polyether	≈ 910 mAh g^−1^ @ 30 cycles	0.2C	[192]

### Electronically Conductive Binders

It is a well established fact that sulfur as well as the insoluble PS discharge products are electronic and ionic insulators, which leads to the utilization of conductive carbon hosts and additives to promote conductivity across Li–S cathodes. Therefore, it is unavoidable that the capacity according to the mass of the entire cathode is reduced, as some of the composite mass goes toward promoting conductivity, while another portion is devoted to the adhesion and structural stability of the electrode (i.e., the binder). If both the conductivity and adhesion could be provided by one electrode component, the mass loading of components, which do not contribute to the capacity of the electrode, can be reduced, thus a higher capacity according to the mass of the entire electrode could be realized. Conductive polymers may be able to fill this requirement with the works toward this aim reviewed below.

**Fig. 30:**
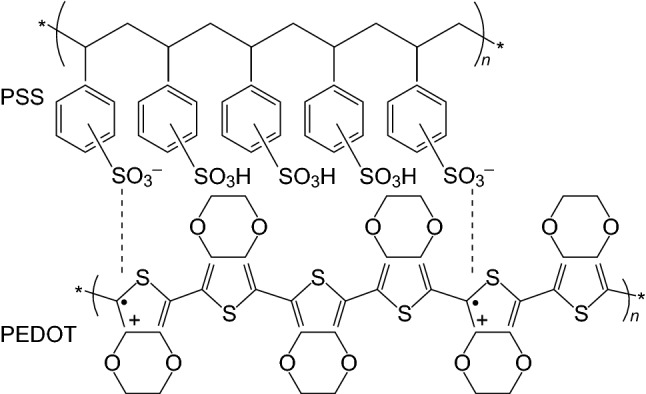
Chemical structure of PEDOT:PSS. Reproduced with permission from Ref. [193]. Copyright 2015 Springer Nature

Poly(3,4-ethylenedioxythiophene) (PEDOT) can either be used directly, or more generally, as a composite polymer with polystyrene sulfonate (PEDOT:PSS), as shown in Fig. 30, PEDOT:PSS consists of conjugated PEDOT with a positive charge and a negatively charged saturated PSS. In terms of practical application, PEDOT:PSS is the most successful conductive polymer, and has found uses in many electrochemical applications [193]. Recently, researchers focusing on Li–S batteries have applied PEDOT and PEDOT:PSS to Li–S cathodes in order to produce electronically conductive polymeric binding frameworks.

Wang et al. [194] first investigated PEDOT as a binder for sulfur cathodes in Li–S batteries. The group examined the electrochemical performance derived from this binder when commercial micrometric sulfur and prepared nanometric sulfur were used as active materials in two electrolyte systems (DOL:DME and PEGDME) and compared the performance obtained with a PVDF binder. The best electrochemical performance was obtained when the cathode framework was synthesized with the commercial micrometric sulfur power and PEDOT binder in a PEGDME electrolyte, which the group ascribed to a reduced polysulfide dissolution and a more viscous electrolyte, which also reduced particle mobility.

Pan et al. [183] investigated a water-soluble PAA/PEDOT:PSS composite binder for Li–S batteries which delivered synergistic functions in high-performance Li–S cells. The PAA binder modified the electrolyte-electrode interface which improved reaction kinetics and also provided electrode adhesion, while the PEDOT:PSS provided chemical anchoring for PS retention as well as allowed for good electronic and ionic conductivity. The group varied the ratio between the multifunctional binder components, and found that a ratio of 2:3 (PAA to PEDOT:PSS) delivered the optimum performance. As a result, the polymeric conductive binding framework enabled an initial discharge capacity of 1121 mAh g^−1^, and a reversible capacity of 833 mAh g^−1^ after 80 cycles at 0.5C.

Later, an ionically cross-linked PEDOT:PSS/Mg^2+^ network binder was developed by Yan et al. [184]. The Mg^2+^ ions interacted with the free SO_2_OH groups on the PSS backbone, which enabled a robust and conductive 3-D network that could better withstand the volume expansion-related stresses that the framework is exposed to during cycling. As a result, the PEDOT:PSS/Mg^2+^ network binder enabled an initial discharge capacity of 1097 mAh g^−1^ with a 74% capacity retention after 250 cycles at 0.5C.

Polyaniline (PANi), in its acid-doped form, is an electronically conductive polymer. However, the brittle PANi chain can hardly accommodate the stresses associated with volume variation of sulfur during cycling. In response to this, extended conducting PANi with good electrical conductivity was developed by Gao et al. [185] through an anion doping strategy. Sulfuric acid was employed to coordinate with the PANi chain in a *m*-cresol solvent to form the extended chain structure, which subsequently enabled a “cobweb” structure that efficiently bonded the active materials with sufficient space for electrolyte swelling and channels for ion transfer, even under an intriguingly low binder dose of 2 wt%. Additionally, the positively charged conductive matrix and the heteroatoms also help to electrostatically and chemically adsorb polysulfides for inhibited shuttling behavior. Owing to these merits, a sulfur electrode based on cobweb PANi binder displayed a reduced internal resistance and faster reaction kinetics, corresponding to a ca. 104% and 74% increase in the specific capacity at a current density of 122 and 610 mA g^−1^, respectively, when compared to the PVDF-based cathode.

Polypyrrole (PPy) is another conductive polymer which has been successfully applied to other LIB systems but has struggled to be implemented in Li–S cells due to its brittleness, making its direct use difficult. To circumvent this, Milroy et al. fabricated a conductive, electroactive, and elastic PPy/polyurethane (PU) multifunctional binder for a free-standing and flexible Li–S cathode [186]. The PPyPU binder delivered dual benefits; an electronically conductive network deriving from the PPy with mechanical pliability from the PU, which can help to accommodate the severe volume change characteristic of sulfur cathodes. A high reversible discharge capacity of ca. 1000 mAh g^−1^ was delivered after 100 cycles at 3C rate owing to the prevention of premature electrode degradation by the PPyPU binder.

**Fig. 31:**
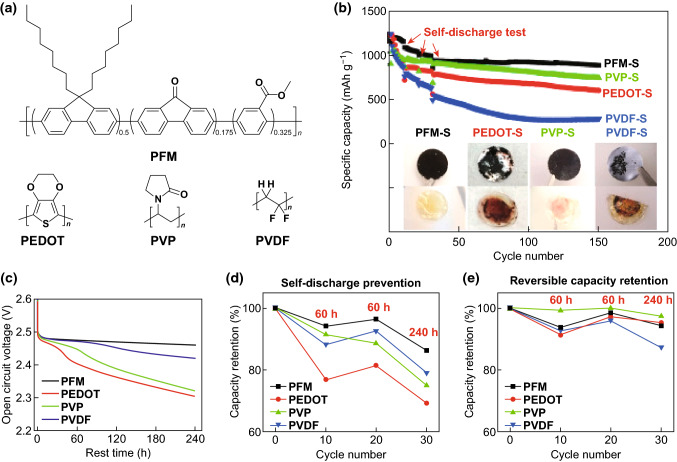
**a** Chemical structures of the four different binders: PFM, PEDOT, PVP, and PVDF. **b** Cycling performance at C/10 and self-discharge performance of cathodes with different binders. **c** The open circuit voltage change versus rest time during the third self-discharge rest of 240 h for the PFM-S, PEDOT-S, PVP-S, and PVDF-S cathodes. The self-discharge capacity prevention ratio (**d**) and reversible capacity retention ratio (**e**) for cathodes with different binders during the self-discharge test. Reproduced with permission from Ref. [187]. Copyright 2015 Elsevier

Poly(9,9-dioctylfluorene-*co*-fluorenone-*co*-methylbenzoic ester) (PFM) binder (Fig. 31a) is a specifically designed polymer with both carbonyl groups for chemical sulfur anchoring and an enhanced electronic conductivity developed by Ai et al. [187]. In their study, the group chose representative polymer binders with specific functionality to compare with the PFM binder. PEDOT:PSS was chosen as an example binder which displays electronic conductivity, PVP was chosen for its chemical PS anchoring ability, and PVDF was chosen as it has neither functionality. Upon investigating the obtained electrochemical performances, it can be noted that between the PEDOT:PSS and PVP-based electrode, the PEDOT:PSS electrode displays a comparatively higher initial discharge capacity but a faster capacity fading upon cycling, whereas the opposite is true for the PVP binder (i.e. a comparatively lower initial capacity but better capacity retention). The group supposed that the electronic conductivity of PEDOT:PSS allowed for a greater degree of initial sulfur utilization, whereas the chemical bonding mediated by the PVP binder resulted in the improved capacity retention. The PFM binder combines both of these traits and as a result delivers the best electrochemical performance. Post-mortem SEM analysis of the top and bottom of the PFM electrodes reveal that in the fully charged state, the PFM binder enables the long-chain PS to be precipitated as elemental sulfur homogeneously throughout the entire electrode owing to combined effects of the carbonyl functional groups and the conductivity of the binder. Complete Li_2_S precipitation is also mediated by the PFM binder owing to the strong affinity between the carbonyl groups and Li_2_S as well as an increased amount of reaction sites for Li_2_S precipitation, owing to the enhanced conductive surface of the multifunctional PFM binding framework.

### Ionically Conductive Binders

Ionically conductive binders can help overcome the low ionic conductivity of sulfur and its discharge product, Li_2_S, so that sulfur utilization and mass transport can be improved within the electrode. The work using ionically conductive binders is reviewed below.

**Fig. 32:**
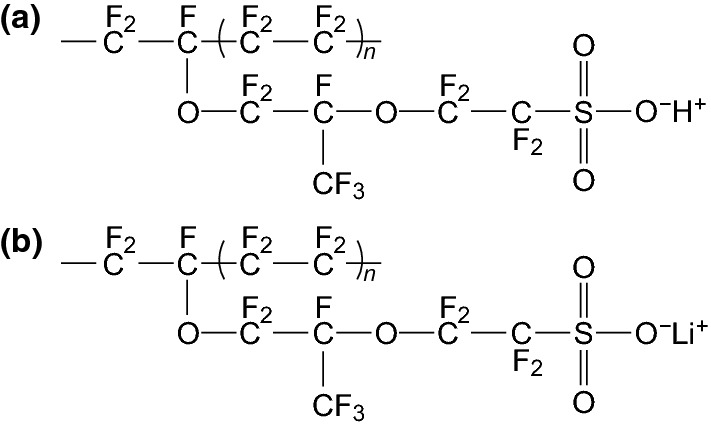
Chemical structures of H-Nafion (**a**) and Li-Nafion (**b**). Reproduced with permission from Ref. [196]. Copyright 2018 Elsevier

One such example is Nafion, a perfluorosulfonate ionomer (ionic polymer) which is most commonly used in proton exchange membranes [195]. The ion conducting properties of Nafion can be altered through cation exchange, as evidenced by Schneider et al. [115]. The group treated the commercial Nafion polymer with LiOH to carry out a cation exchange and examined the material as a binder for Li–S batteries (Fig. 32). Electrodes were fabricated using the Li-Nafion as a binder with an additional Li-Nafion layer spray coated on the surface. The resultant batteries displayed an improved initial discharge capacity when compared to CMC and PTFE based cells, which indicates an improved sulfur utilization owing to the improved ionic conductivity of the Li-Nafion based cell.

Following this, Li et al. combined Li-Nafion, PVP and nanosilica as a multifunctional binder for high-performance Li–S batteries [188]. Each component of the binder contributed to improved cell performance. The Li-Nafion improved Li^+^ supply for sulfur redox reactions. The PVP provided PS anchoring for a reduced shuttle effect, improved the mechanical properties of the composite, and enabled a good dispersion of active materials within the sulfur electrode. The impregnated nanosilica could provide further inhibit the shuttle effect due to the strong affinity toward its polar surface and polysulfides, while simultaneously introducing abundant interfaces within the electrode for improved electrolyte wetting. Attributed to these favorable functionalities, the sulfur electrode based on the composite binder achieved a high sulfur utilization with initial discharge capacity of 1373 mAh g^−1^ at 0.2C, excellent sulfur redox kinetics with highly reversible capacity of 470 mAh g^−1^ at a high current rate up to 5C, and superb cycling stability over 300 cycles at 1C. More recently, Gao et al. used a Li-Nafion resin as both the binder and solid electrolyte in Li–S cells [196]. An optimized loading of 40% Li-Nafion and 10% conductive additive allowed for a balance of ionic and electronic conductivity in the cathode, which delivered a reversible capacity of 895 mAh g^−1^ at 1C with an 89% capacity retention after 100 cycles.

Cheng et al. developed a sulfonated poly (ether ether ketone) (SPEEK) polymer as functional binder for sulfur electrode [189]. The ether and benzene rings endowed the SPEEK with an appropriate combination of flexibility and stiffness, leading to good adhesion for electrode active materials, while the abundant carbonyl, sulfonyl, and benzene ring groups contributed to a strong electronegativity that repelled the dissolution and diffusion of polysulfide anions, thus facilitating the inhibition on polysulfide shuttling. As a result, the SPEEK based Li–S cell delivered a more stable performance after 300 cycles at a current density of 1000 mA g^−1^ compared with the PVDF-based cell.

As mentioned earlier, the mechanical strength and adhesive properties of PEO frameworks suffer from swelling/dissolution in organic electrolytes. Zhang et al. attempted to rectify this phenomenon by creating a 3D-cross-linked tannic acid (TA)/PEO binder with enhanced ionic conductivity for Li–S cells [190]. The formation of the 3D network was realized through hydrogen bonding interactions between the TA and the PEO which could enable the TA/PEO framework to provide strong adhesion even after submersion in the electrolyte. Electrochemical investigation revealed that the TA/PEO framework delivered a stable discharge capacity of 476.7 mAh g^−1^ after an outstanding 1000 cycles, owing to the composite network binder’s PS anchoring ability and mechanical properties. Post-mortem SEM analysis of the cathodes revealed that the PEO- and PVDF-based electrodes displayed a thick Li_2_S layer deposited on the surface, whereas the TA/PEO electrode had a relatively uniform distribution of discharge products, which the authors suggest was due to a facile diffusion of lithium ions throughout the framework.

### Redox-Active Binders

A simple and straightforward strategy to achieve a reactive binder is incorporating active sulfur into the binder structure, which can contribute additional capacity while maintaining good electrode integrity. Trofimov et al. [197] prepared bis-[3-(vinyloxyethoxy)-2-hydroxypropyl-] polysulfides (BVPS) by reacting ethylene glycol vinyl glycidyl ether (EGVGE) with Na_2_S_4_ in the presence of NaHCO_3_ and a phase transfer catalyst triethylbenzylammonium chloride. The obtained BVPS contained 24.5% sulfur (*n* = 2, 3, where n represents the length of the polysulfide chain in the BVPS molecule) bridging the symmetric organic moieties, which was further copolymerized with elemental sulfur at 130 °C for 1 h to yield a polymer containing up to 32.6% sulfur (*n* = 4). The polymerization leads to the formation of cross-linked polymers, which were used as the active binder for Li–S batteries. The obtained binder exhibited strong adhesion that was able to retain a robust electrode even under low binder content of 5%. Meanwhile, the binder also contributed additional capacity due to the redox reactivity of the sulfur incorporated in the binder structure.

Imide-based organic compounds have recently been investigated as redox-active mediators in Li–S systems by Frischmann et al. [198]. The group then implemented *π*-stacked perylene bisimide (PBI) as redox-active supramolecular polymer binders in order to overcome the ionic and electronic bottlenecks in sulfur cathodes [191]. The PBI binder offered self-healing properties which could reduce structural damage from the active material volume expansion upon cycling. By fabricating a PBI/PVDF composite binder, the over-potential of the electrodes during discharge was minimized, as evidenced by a galvanostatic intermittent titration technique (GITT). The group then went on to further investigated a lithiated, redox-active, aqueous-soluble PBI binder which showed further electrochemical improvements [199].

**Fig. 33:**
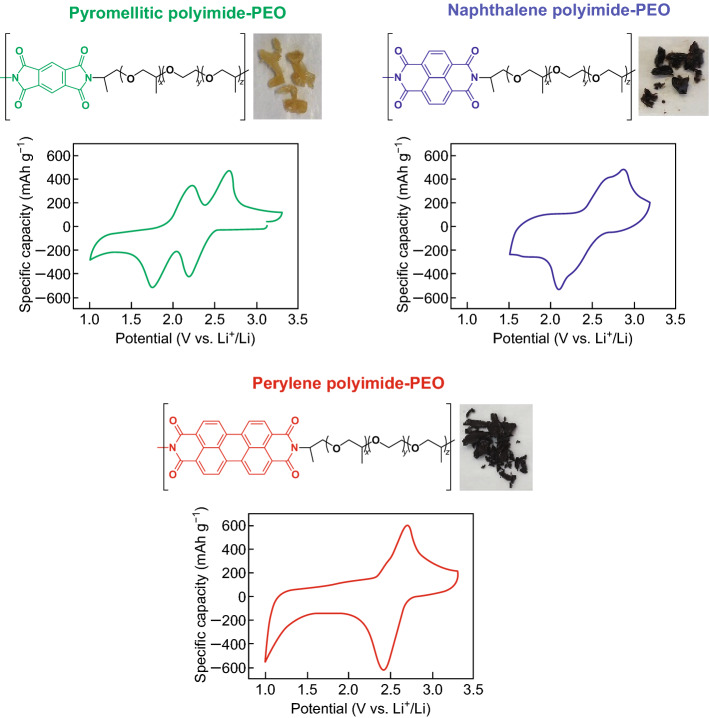
Chemical structure, physical aspect and cyclic voltammogram of polyimide–polyether copolymers. Reproduced with permission from Ref. [192]. Copyright 2017 Elsevier

Hernández et al. [192] investigated three polyimide–polyether composite redox-active binders for Li–S batteries (Fig. 33). Among the pyromellitic, naphthalene, and perylene polyimides, the cell based on the naphthalene–polyether composite binder showed a higher sulfur utilization and a lower polarization, and thus delivered the best electrochemical performance for a few reasons. The redox potential of the naphthalene–polyether coincided best with the sulfur redox potential, and, as a result, successfully facilitated charge transfer across the binding framework and sulfur interfaces in turn improving active mass utilization. The incorporation of PEO within the composite increased the solubility of the copolymer, making electrode fabrication easier, and during cycling enabled an improved mass transport across the electrode while simultaneously limiting PS diffusion from the cathode. The resultant naphthalene–polyether based electrodes delivered an initial capacity of 1300 mAh g^−1^ with a 70% capacity retention after 30 cycles at 0.2C.

Overall, multifunctional PCFs can increase the performance of a sulfur cathode relative to its entire mass by endowing a cell component which would have otherwise not contributed to the electrochemical function of a cell (beyond providing structural stability) with such abilities as electronic and ionic conductivity or redox activity. Further improvements could be achieved by combining these multifunctional binders with relevant sulfur hosts for an increased performance.

## Conclusions

Thus far, the majority of efforts to address the inherent challenges of Li–S batteries have been focused toward the design of micro-, nano-, or molecular structured sulfur hosts. The function of binders and the widespread availability of multifunctional binders has been neglected. Recently, sulfur host-based cathodes which utilize the traditional PVDF binder have been the dominant research direction, however the role novel binders play in these cathodes is beginning to be explored. Briefly, by the careful selection of multifunctional binders and sulfur hosts, the following benefits could be realized:

1. By combining natural polymers with hosts synthesized via green chemical routes, the overall environmental impact of Li–S cell fabrication could be reduced.
2. By combining synthetic binders, cross-linked binders, or composite binders with a suitable sulfur host, further improvements in sulfur loading, sulfur utilization, E/S ratio, and safety of the Li–S system could be achieved.
3. By using relevant multifunctional binders, the specific capacity of the Li–S cathode could be improved by reducing the amount of electrochemically inactive components.

These relatively new research directions could provide vast improvements in the future, although in the case of host-free PCFs, special attention must be paid to assure that the polymeric binder can not only initiate strong adhesive forces between the electrode components, it must also be able to retain a stable void structure during sulfur dissolution. In short, binder research in Li–S batteries is an under explored and fruitful research direction.
